# Supplementary material for: Trycycler: consensus long-read assemblies for bacterial genomes
Source: Genome Biol. 2021 Sep 14;22:266. doi: 10.1186/s13059-021-02483-z (PMC8442456; doi:10.1186/s13059-021-02483-z)
Supplement: Supplementary file 1 — Additional file 1. Supplementary Figs. S1–S7. [file 13059_2021_2483_MOESM1_ESM.pdf]

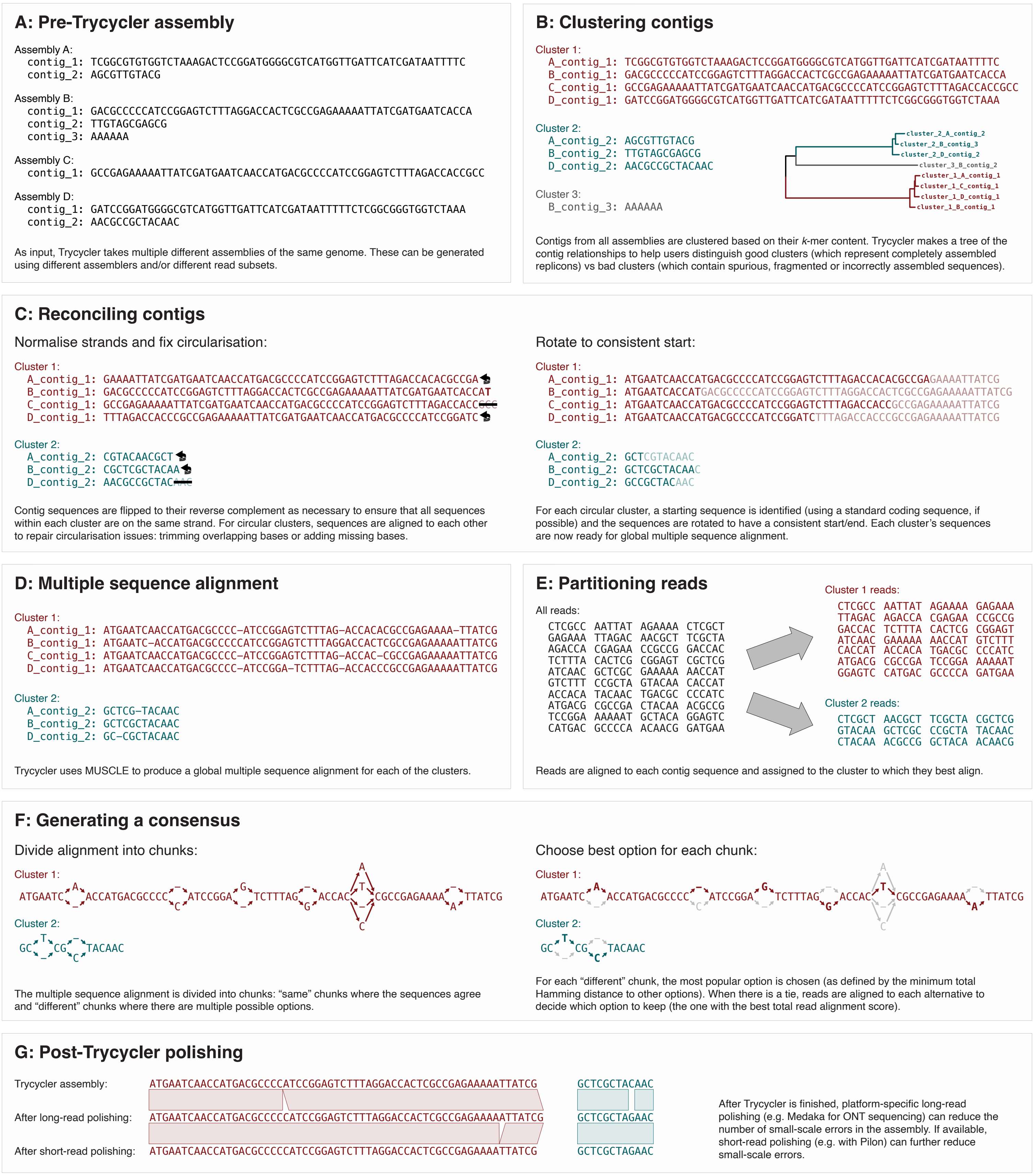

Fig. S1: steps in the Trycycler assembly pipeline.

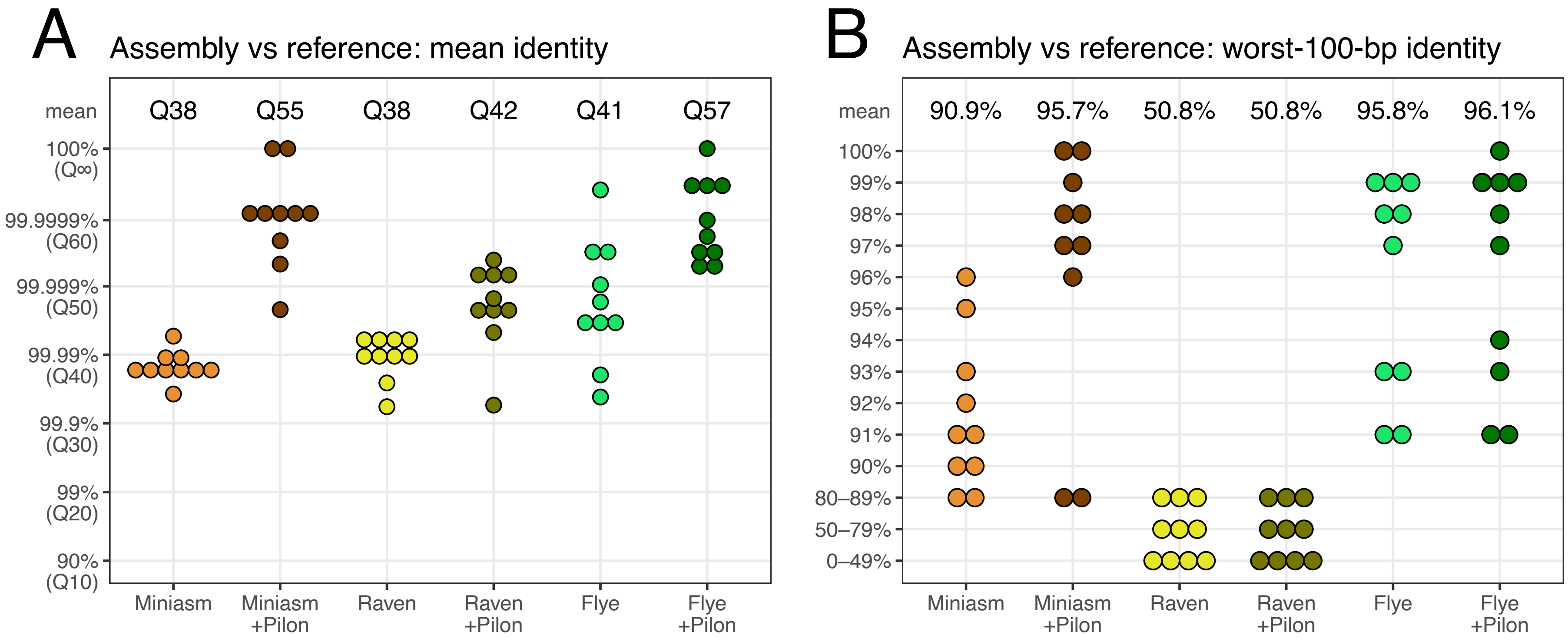

Fig. S2: results for the simulated read tests for long-read-only assemblers. This figure contains the same analyses as are shown in Figure 2, but it includes assemblies from all long-read-only assemblers (both before and after Pilon polishing).

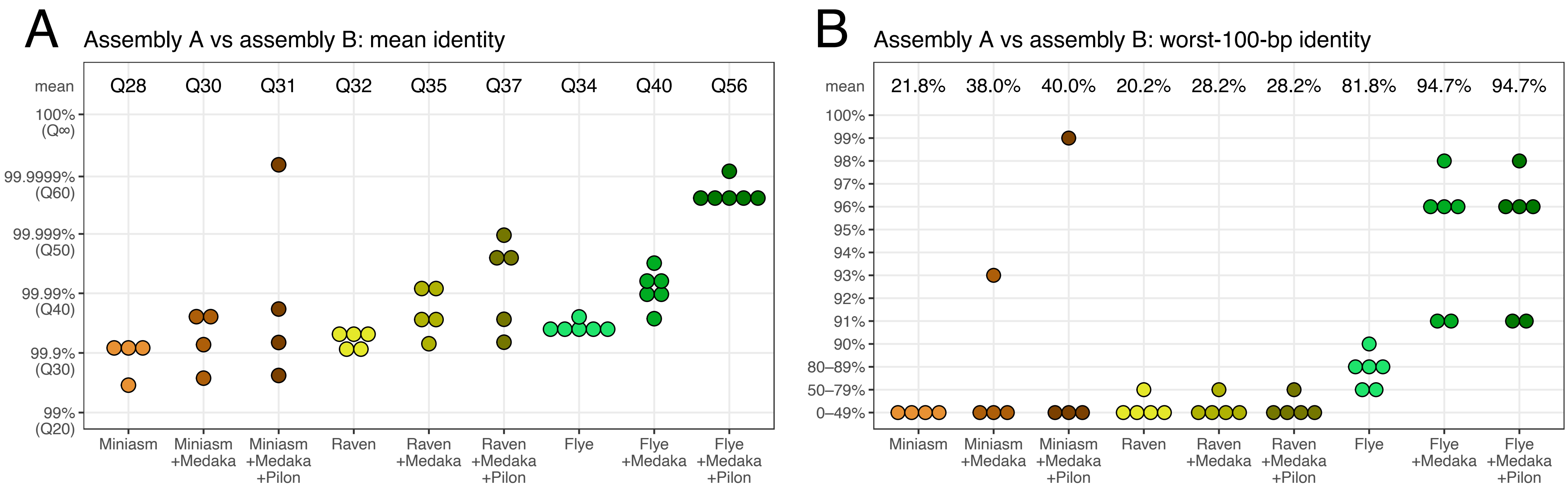

Fig. S3: results for the real read tests for long-read-only assemblers. This figure contains the same analyses as are shown in Figure 3, but it includes assemblies from all long-read-only assemblers (unpolished, Medaka-polished and Medaka+Pilon-polished for each). In two of the six genomes, Miniasm failed to produce a completed chromosome for both read sets, resulting in only four data points. In one of the six genomes, Raven failed to produce a completed chromosome for both read sets, resulting in only five data points.

Campylobacter jejuni (simulated reads)

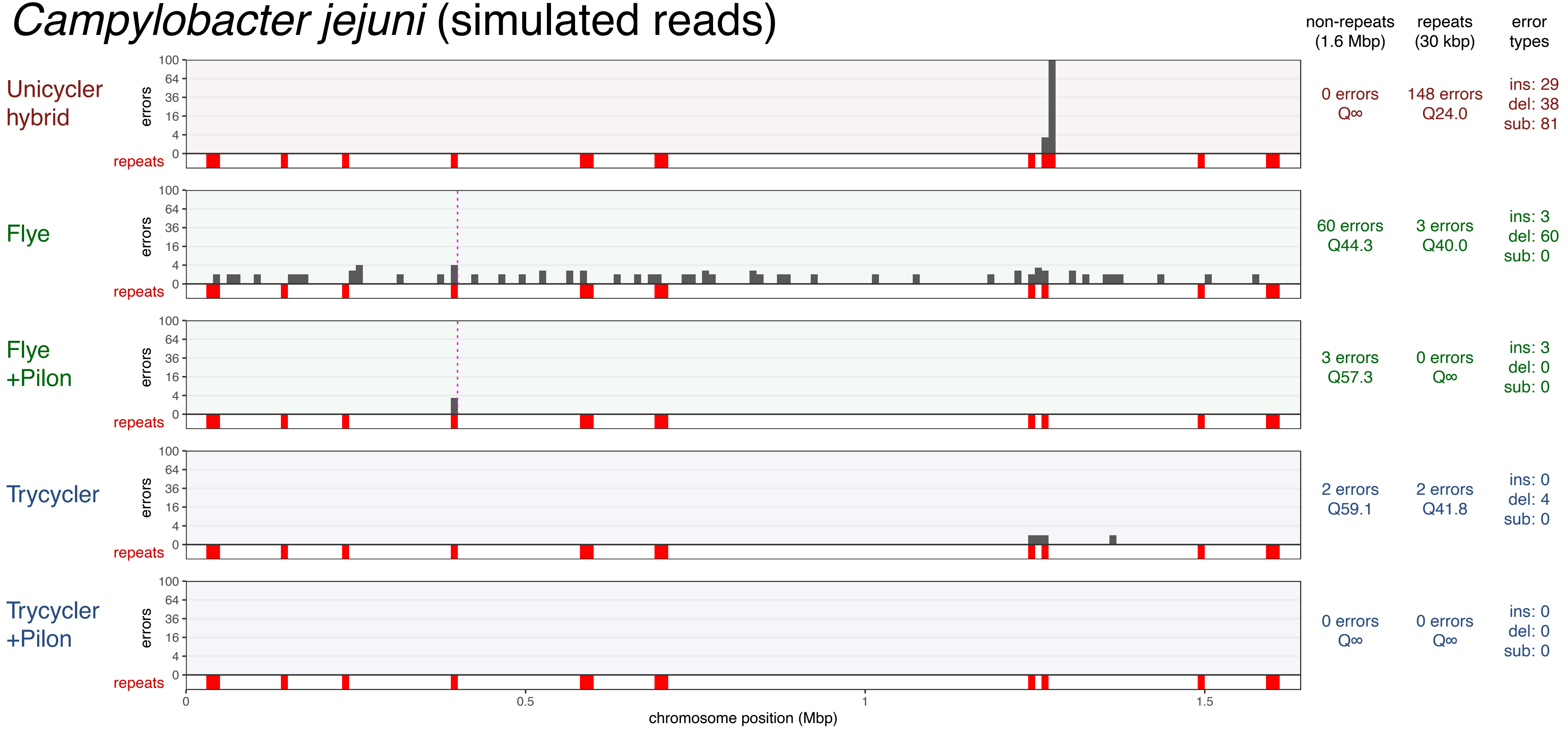

Fig. S4-a: error distributions (as defined by alignment to the reference sequence) for the simulated-read assemblies of the *Campylobacter jejuni* chromosome. Repetitive regions of the genome are shown as red blocks at the bottom of each plot. For Flye assemblies, the contig start/end position (where the sequence began before it was rotated to be consistent with the reference) is shown by the dashed line.

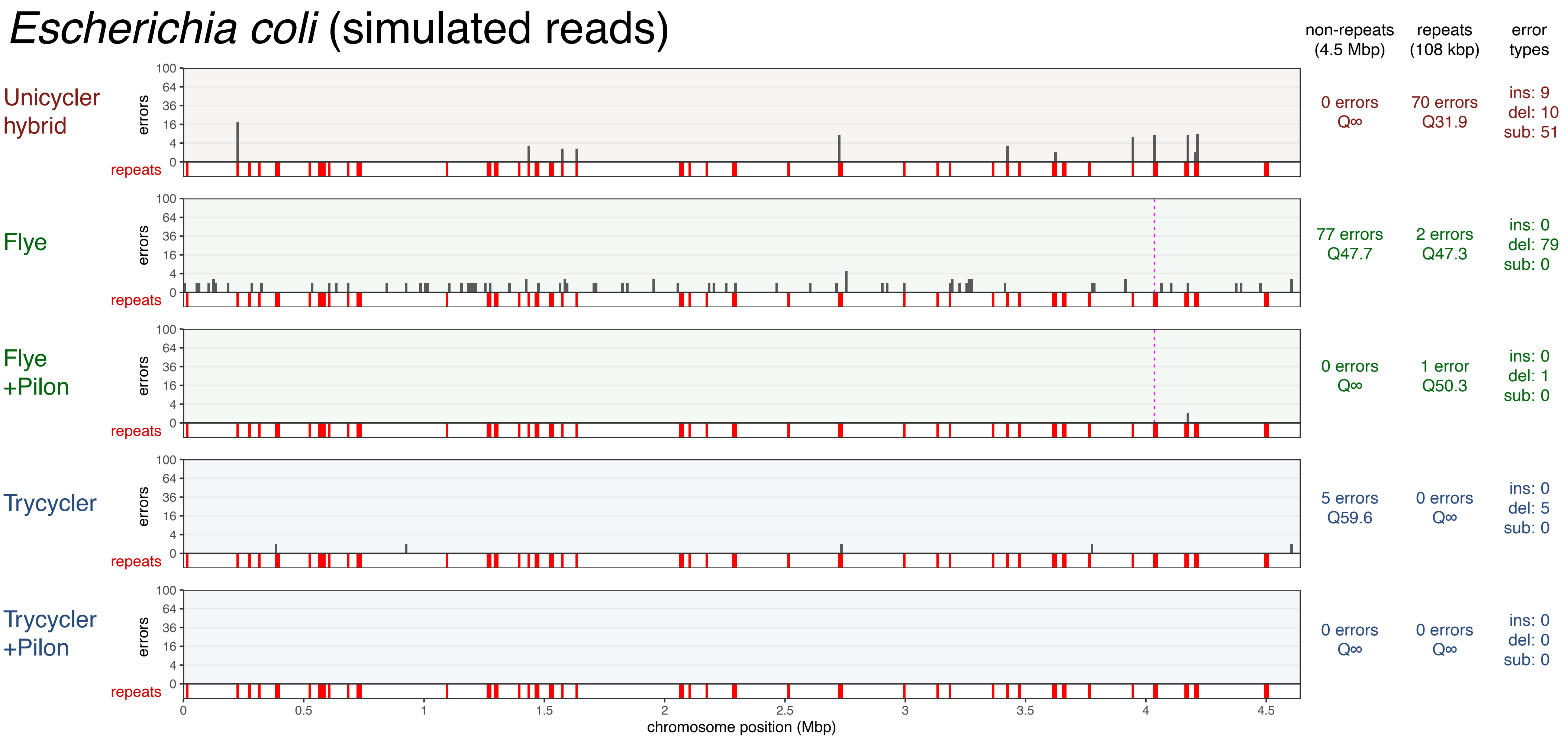

Fig. S4-b: error distributions (as defined by alignment to the reference sequence) for the simulated-read assemblies of the *Escherichia coli* chromosome. Repetitive regions of the genome are shown as red blocks at the bottom of each plot. For Flye assemblies, the contig start/end position (where the sequence began before it was rotated to be consistent with the reference) is shown by the dashed line.

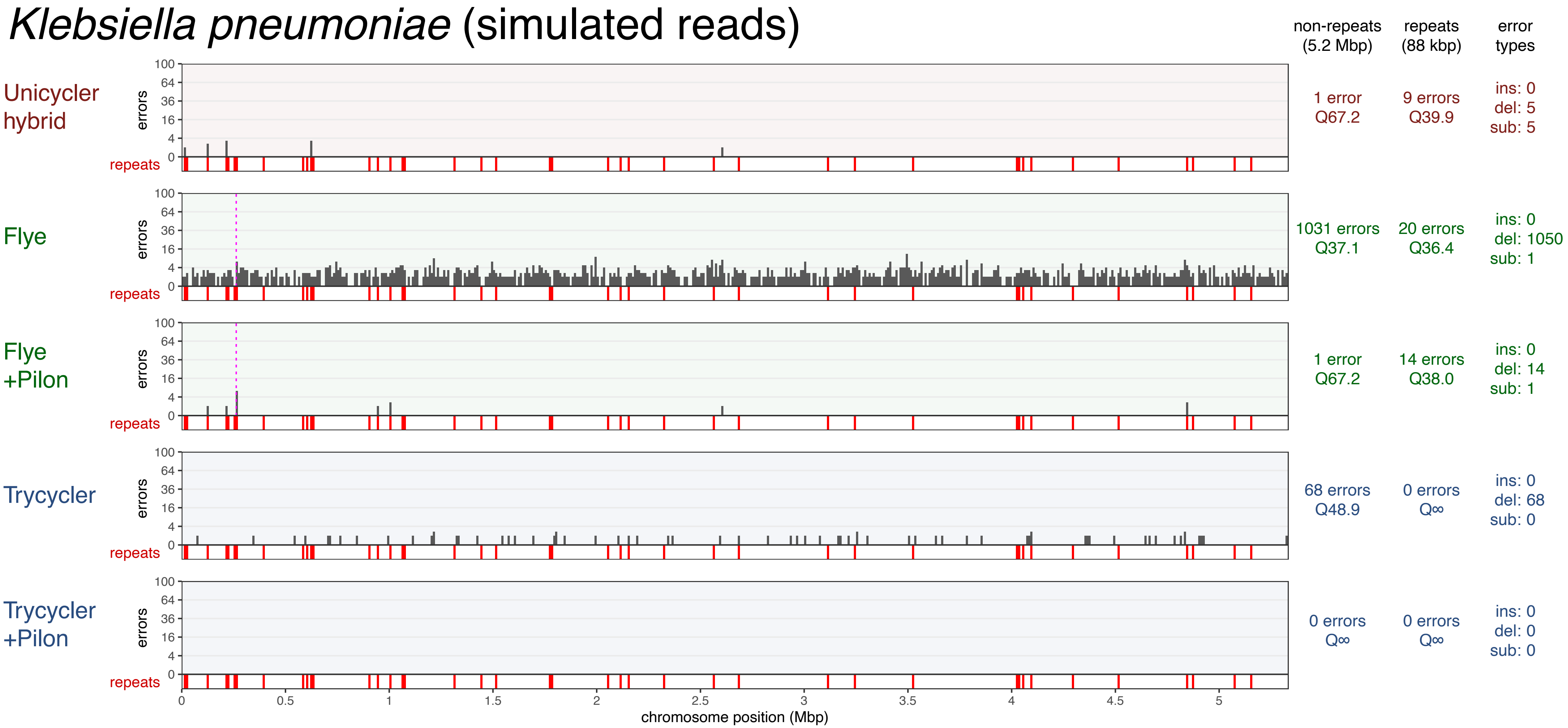

Fig. S4-c: error distributions (as defined by alignment to the reference sequence) for the simulated-read assemblies of the *Klebsiella pneumoniae* chromosome. Repetitive regions of the genome are shown as red blocks at the bottom of each plot. For Flye assemblies, the contig start/end position (where the sequence began before it was rotated to be consistent with the reference) is shown by the dashed line.

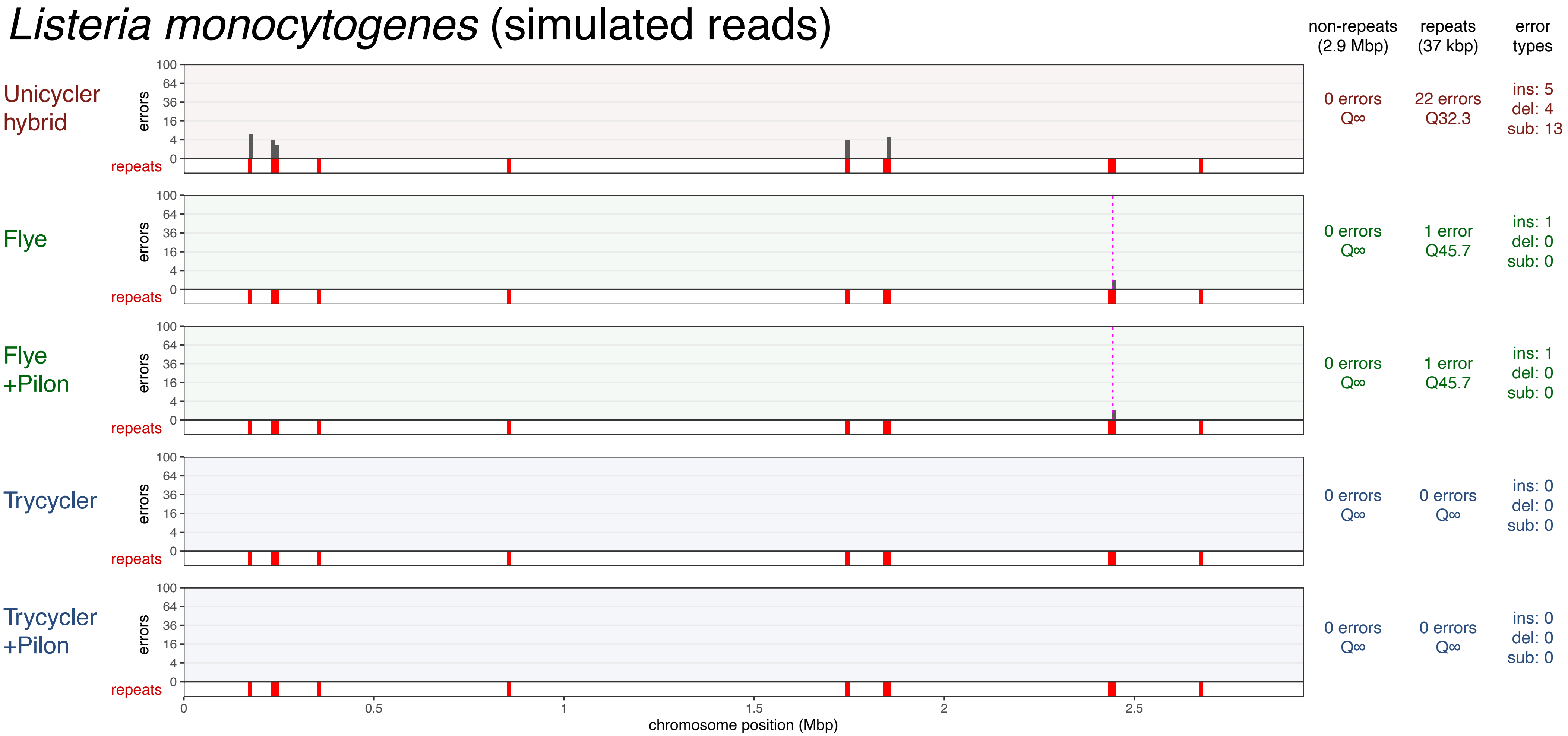

Fig. S4-d: error distributions (as defined by alignment to the reference sequence) for the simulated-read assemblies of the *Listeria monocytogenes* chromosome. Repetitive regions of the genome are shown as red blocks at the bottom of each plot. For Flye assemblies, the contig start/end position (where the sequence began before it was rotated to be consistent with the reference) is shown by the dashed line.

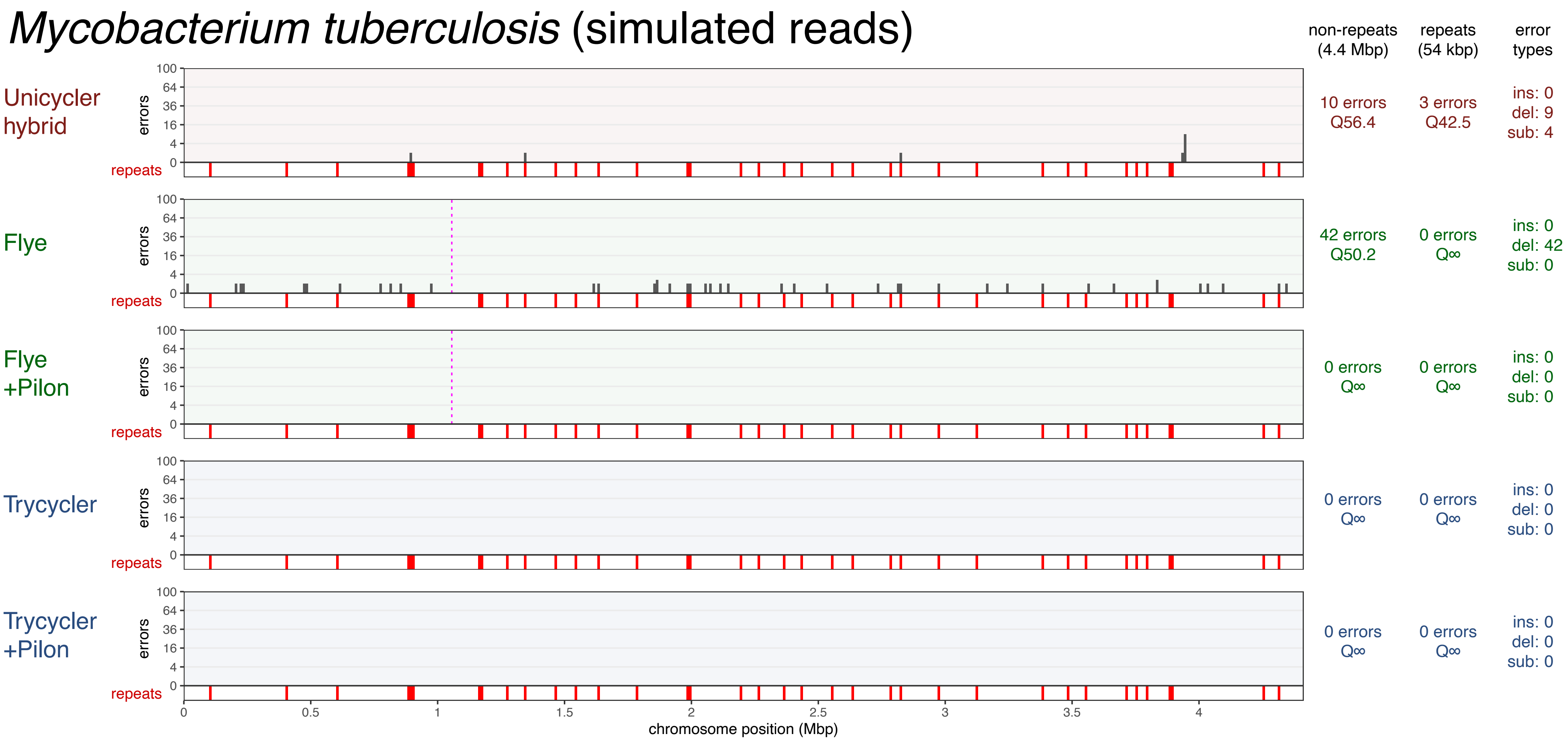

Fig. S4-e: error distributions (as defined by alignment to the reference sequence) for the simulated-read assemblies of the *Mycobacterium tuberculosis* chromosome. Repetitive regions of the genome are shown as red blocks at the bottom of each plot. For Flye assemblies, the contig start/end position (where the sequence began before it was rotated to be consistent with the reference) is shown by the dashed line.

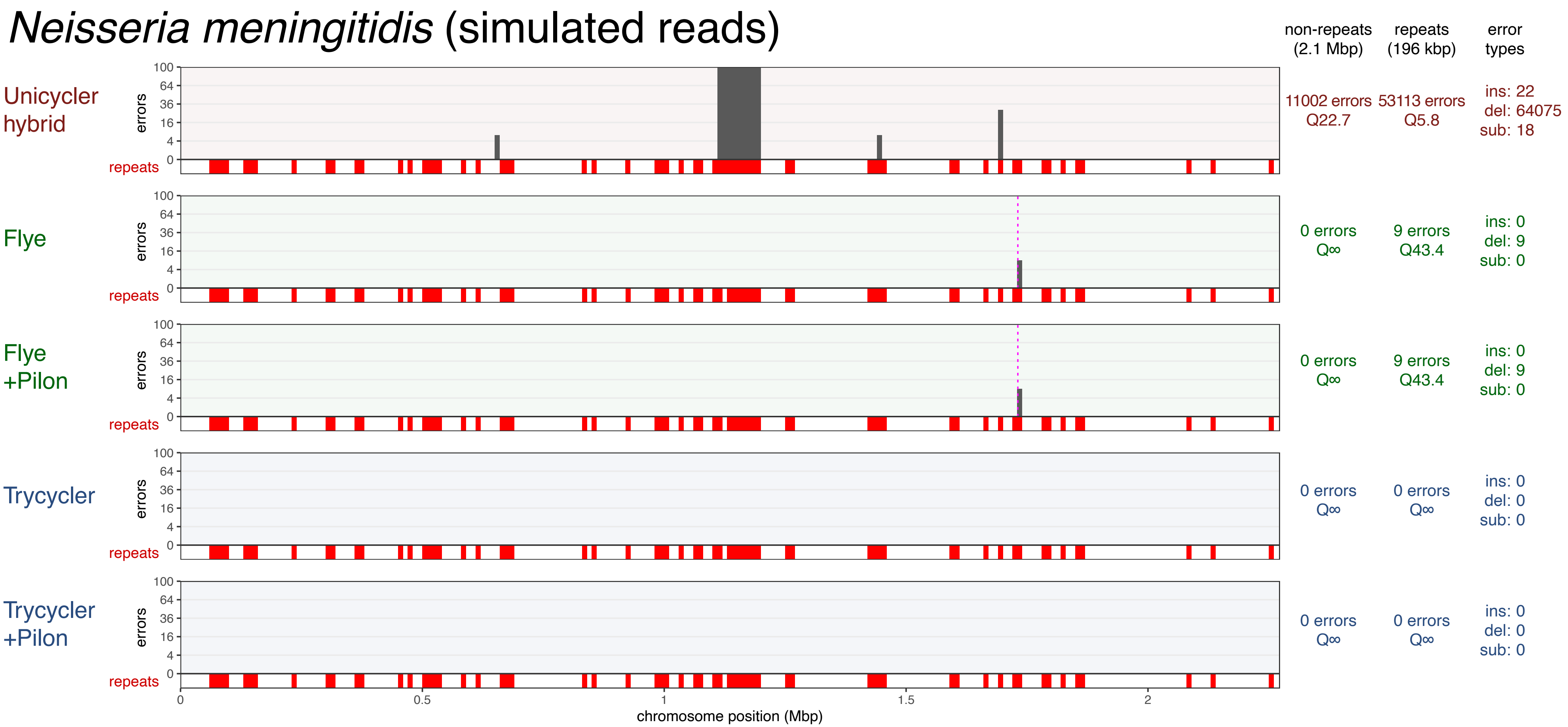

Fig. S4-f: error distributions (as defined by alignment to the reference sequence) for the simulated-read assemblies of the *Neisseria meningitidis* chromosome. Repetitive regions of the genome are shown as red blocks at the bottom of each plot. For Flye assemblies, the contig start/end position (where the sequence began before it was rotated to be consistent with the reference) is shown by the dashed line.

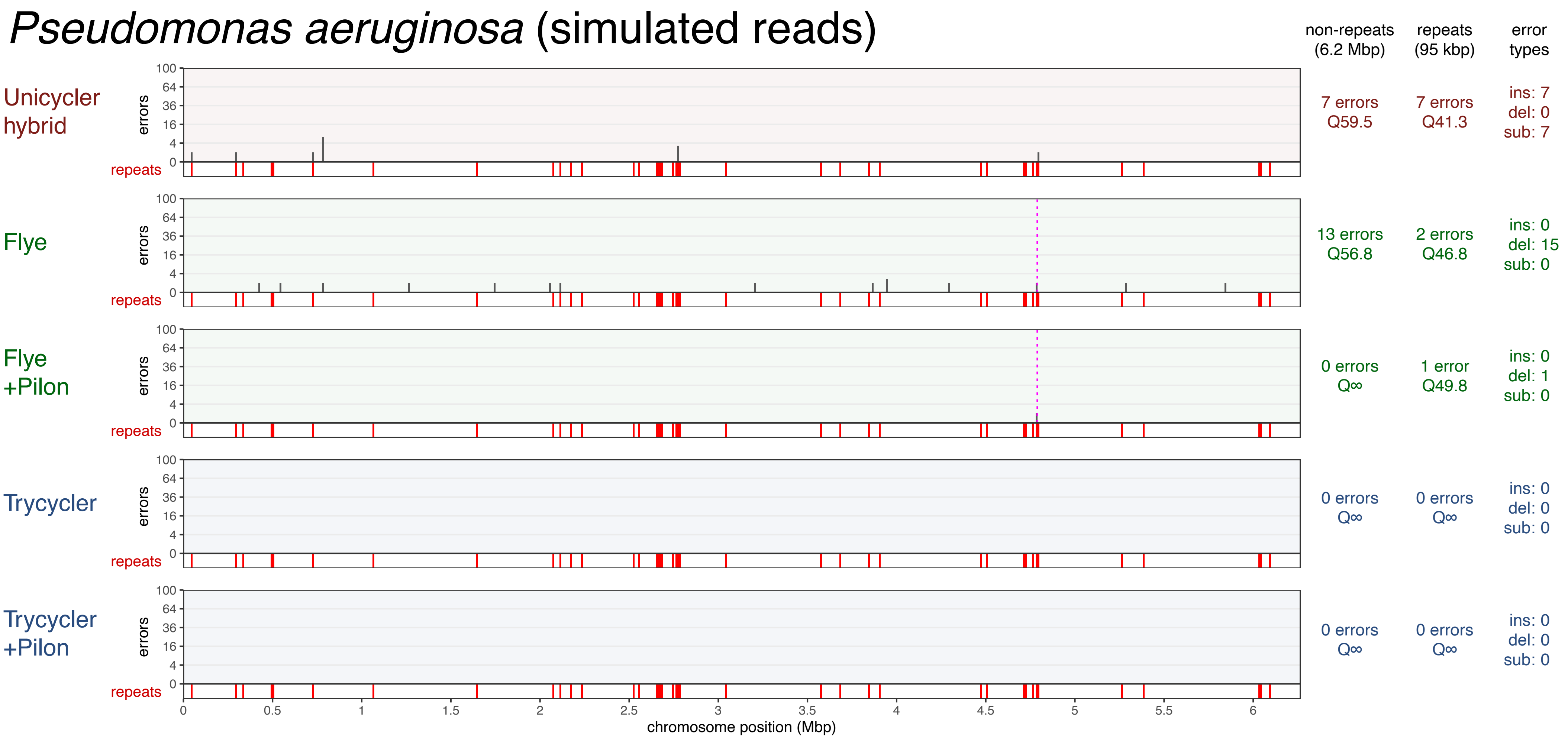

Fig. S4-g: error distributions (as defined by alignment to the reference sequence) for the simulated-read assemblies of the *Pseudomonas aeruginosa* chromosome. Repetitive regions of the genome are shown as red blocks at the bottom of each plot. For Flye assemblies, the contig start/end position (where the sequence began before it was rotated to be consistent with the reference) is shown by the dashed line.

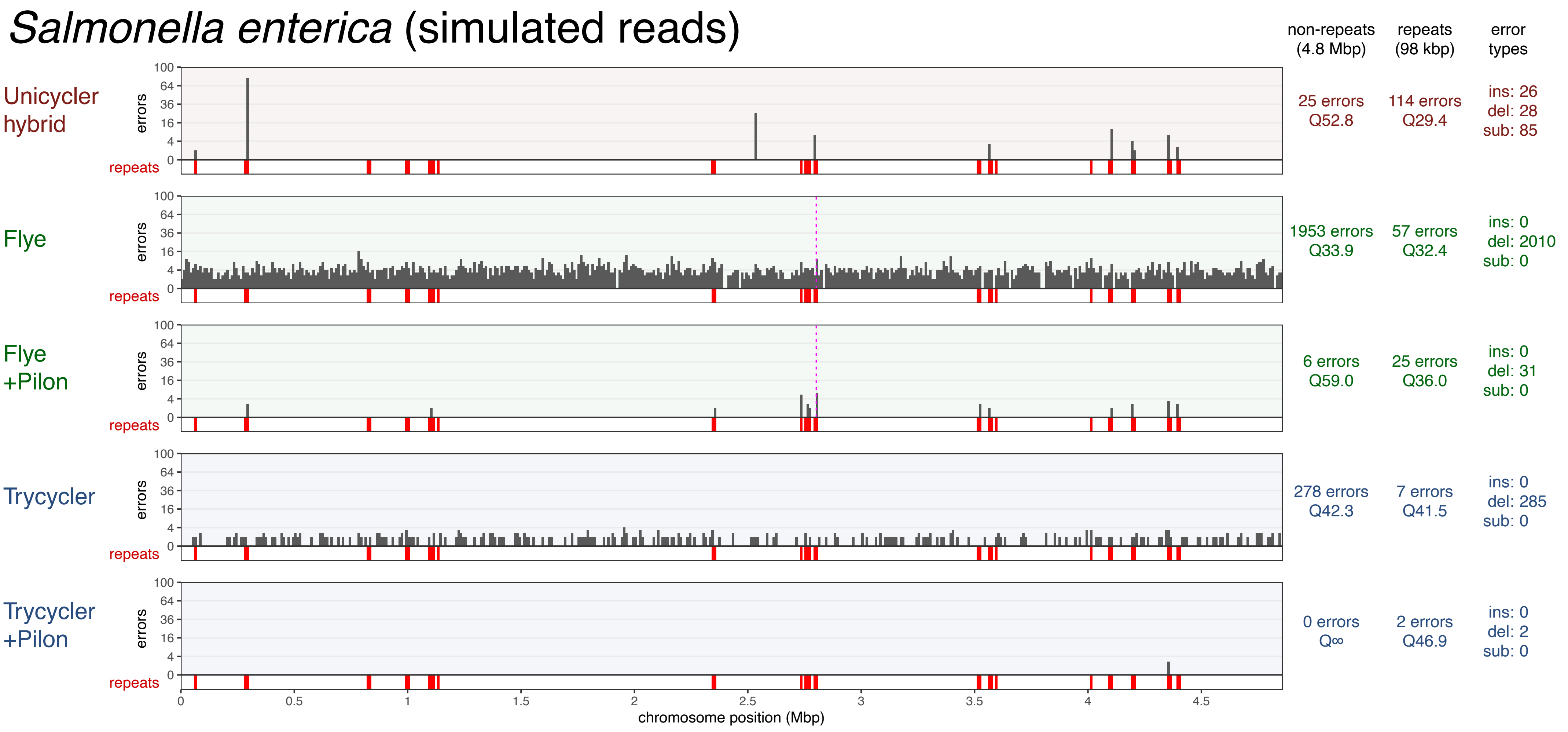

Fig. S4-h: error distributions (as defined by alignment to the reference sequence) for the simulated-read assemblies of the *Salmonella enterica* chromosome. Repetitive regions of the genome are shown as red blocks at the bottom of each plot. For Flye assemblies, the contig start/end position (where the sequence began before it was rotated to be consistent with the reference) is shown by the dashed line.

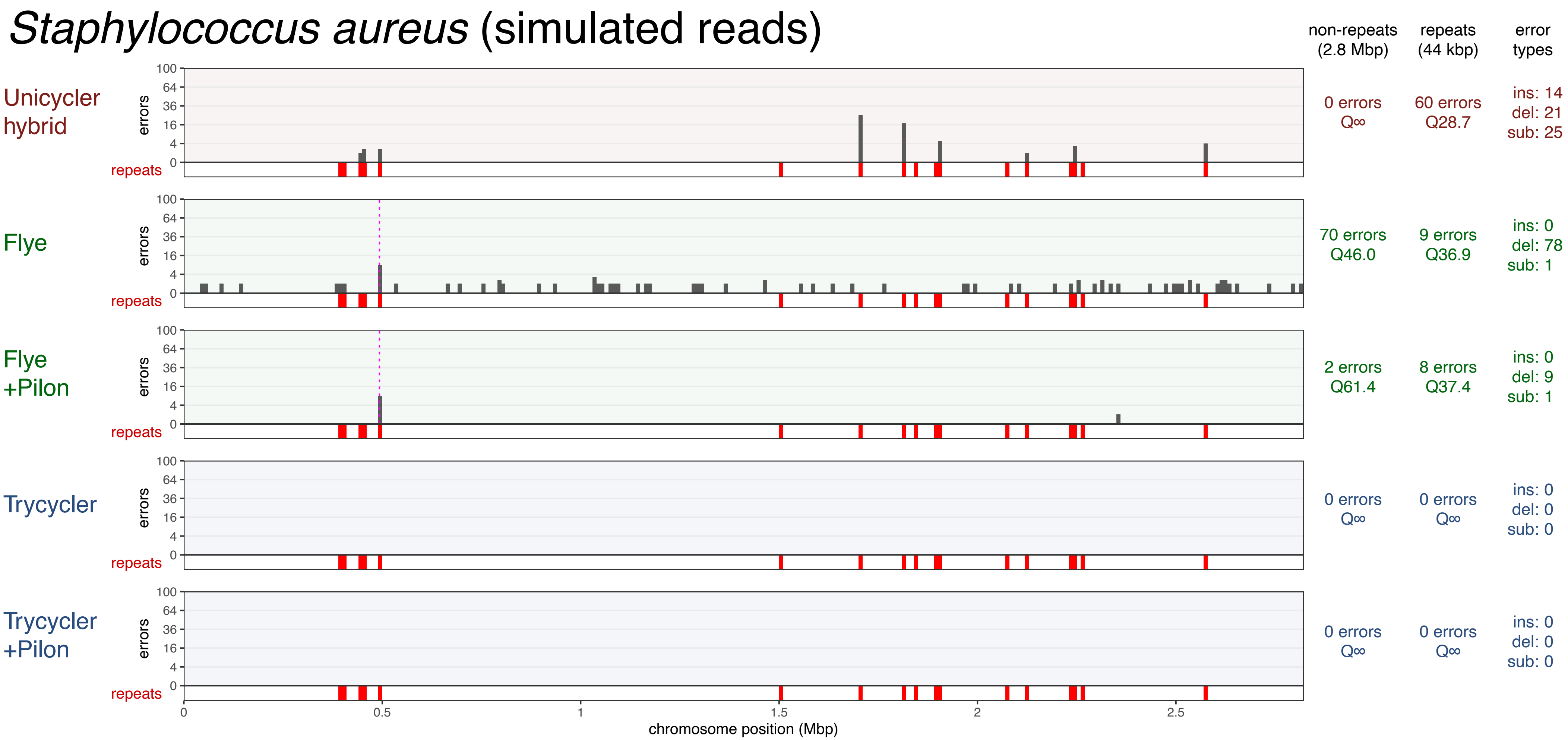

Fig. S4-i: error distributions (as defined by alignment to the reference sequence) for the simulated-read assemblies of the *Staphylococcus aureus* chromosome. Repetitive regions of the genome are shown as red blocks at the bottom of each plot. For Flye assemblies, the contig start/end position (where the sequence began before it was rotated to be consistent with the reference) is shown by the dashed line.

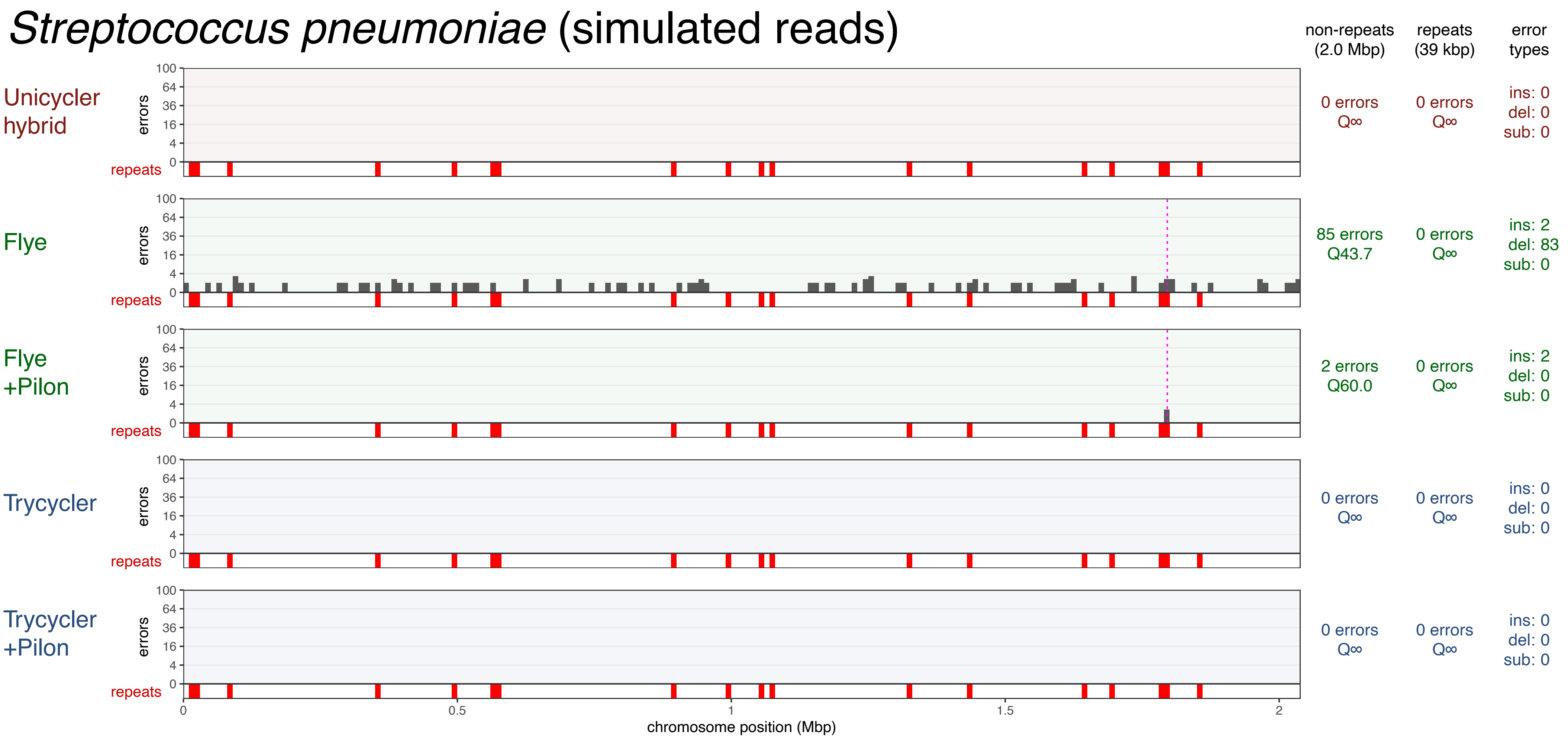

Fig. S4-j: error distributions (as defined by alignment to the reference sequence) for the simulated-read assemblies of the *Streptococcus pneumoniae* chromosome. Repetitive regions of the genome are shown as red blocks at the bottom of each plot. For Flye assemblies, the contig start/end position (where the sequence began before it was rotated to be consistent with the reference) is shown by the dashed line.

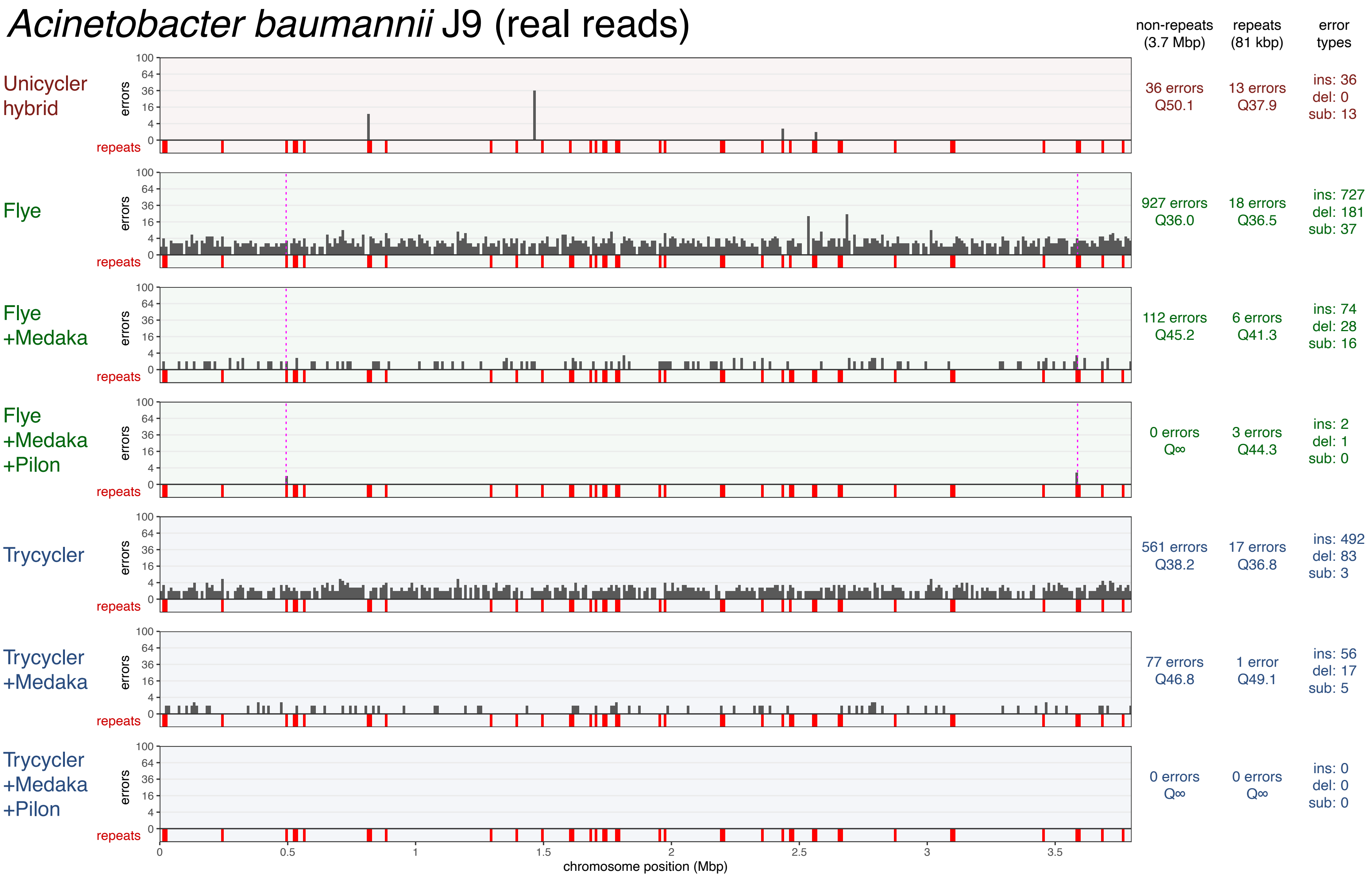

Fig. S4-k: error distributions (as defined by alignment between two independent assemblies) for the real-read assemblies of the *Acinetobacter baumannii* chromosome. Repetitive regions of the genome are shown as red blocks at the bottom of each plot. For Flye assemblies, the contig start/end positions (where the sequences began before they were rotated to be consistent with the reference) are shown by the dashed lines.

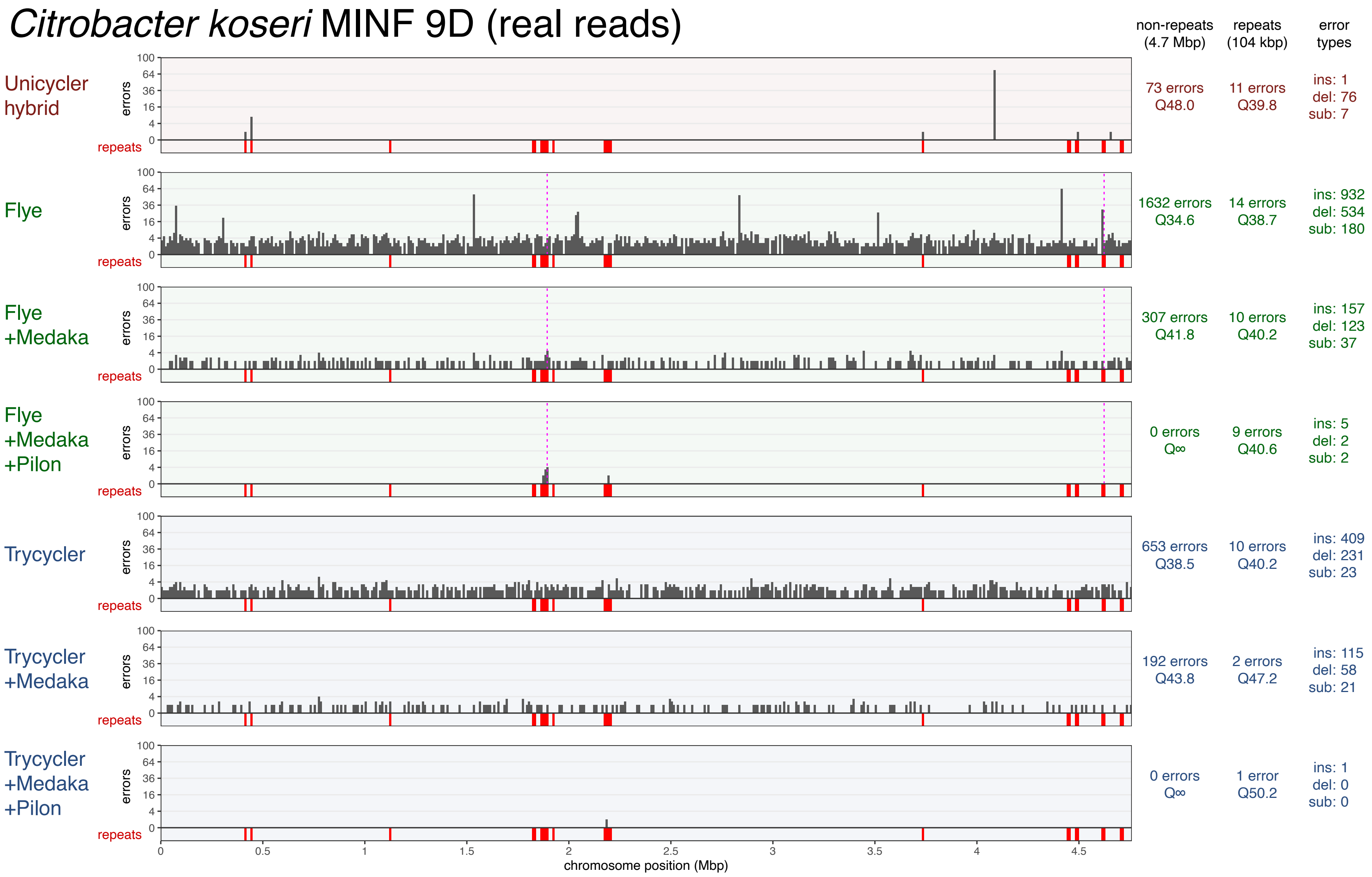

Fig. S4-l: error distributions (as defined by alignment between two independent assemblies) for the real-read assemblies of the *Citrobacter koseri* chromosome. Repetitive regions of the genome are shown as red blocks at the bottom of each plot. For Flye assemblies, the contig start/end positions (where the sequences began before they were rotated to be consistent with the reference) are shown by the dashed lines.

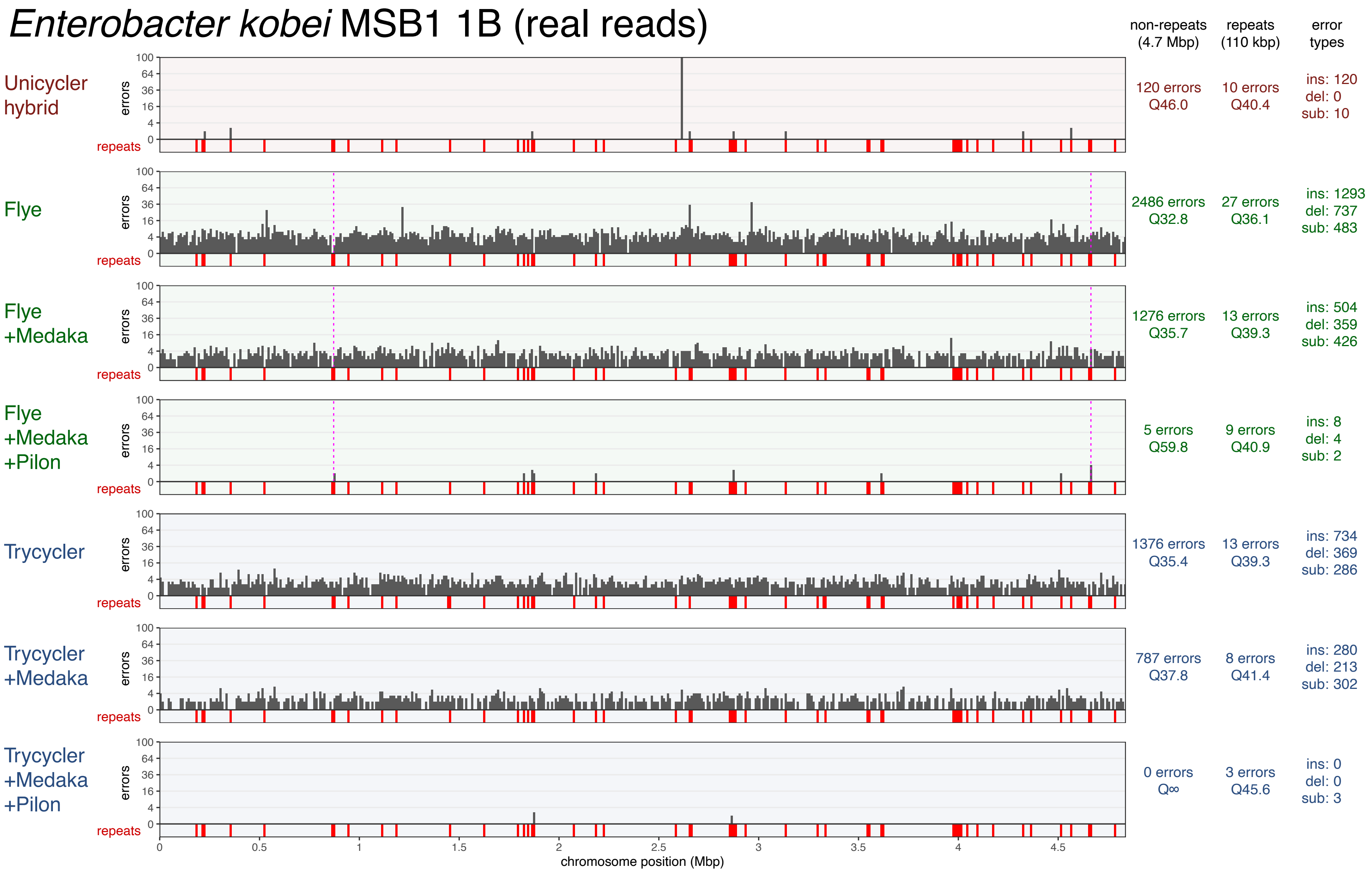

Fig. S4-m: error distributions (as defined by alignment between two independent assemblies) for the real-read assemblies of the *Enterobacter kobei* chromosome. Repetitive regions of the genome are shown as red blocks at the bottom of each plot. For Flye assemblies, the contig start/end positions (where the sequences began before they were rotated to be consistent with the reference) are shown by the dashed lines.

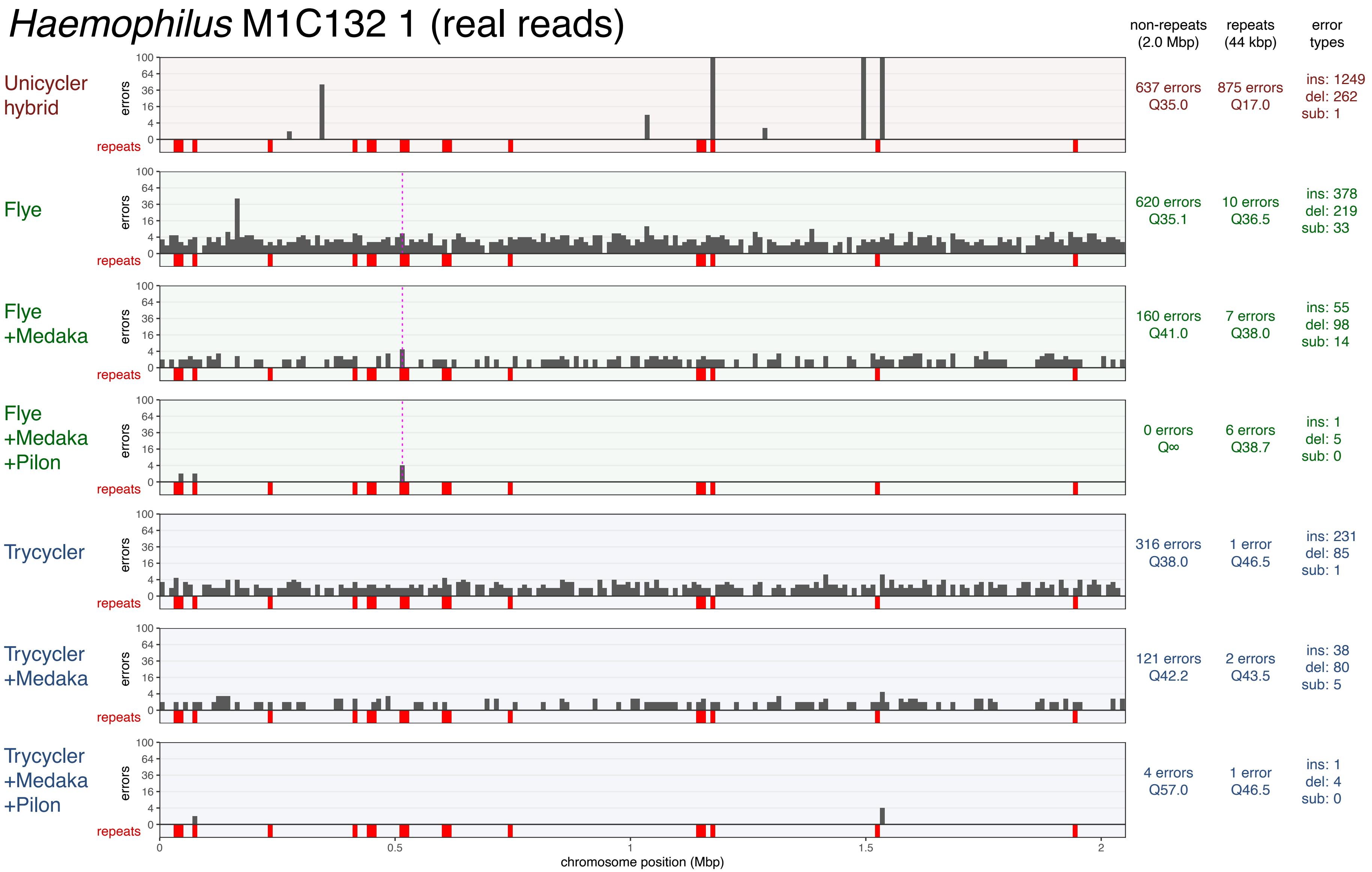

Fig. S4-n: error distributions (as defined by alignment between two independent assemblies) for the real-read assemblies of the *Haemophilus* chromosome. Repetitive regions of the genome are shown as red blocks at the bottom of each plot. For Flye assemblies, the contig start/end positions (where the sequences began before they were rotated to be consistent with the reference) are shown by the dashed lines.

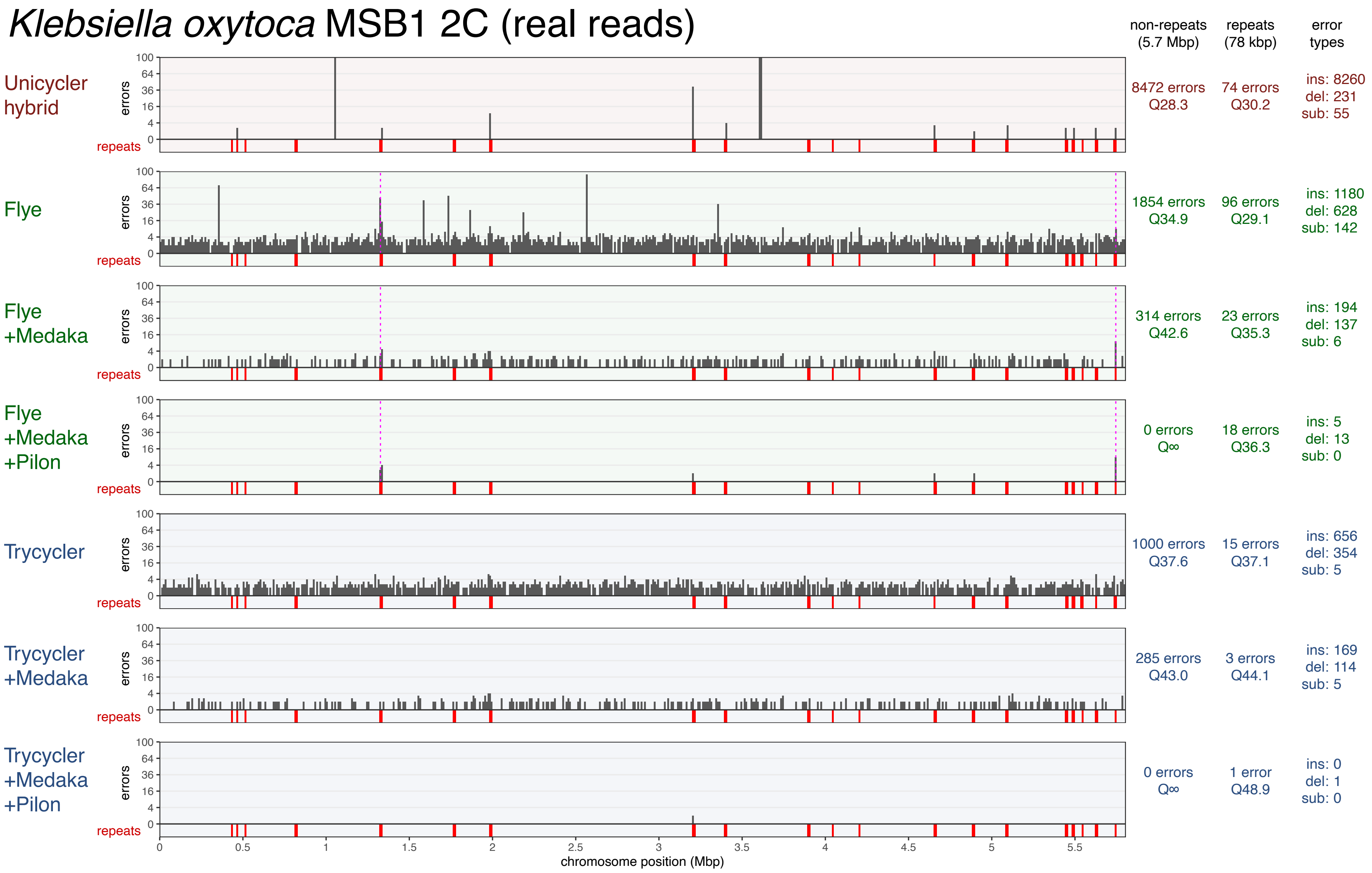

Fig. S4-o: error distributions (as defined by alignment between two independent assemblies) for the real-read assemblies of the *Klebsiella oxytoca* chromosome. Repetitive regions of the genome are shown as red blocks at the bottom of each plot. For Flye assemblies, the contig start/end positions (where the sequences began before they were rotated to be consistent with the reference) are shown by the dashed lines.

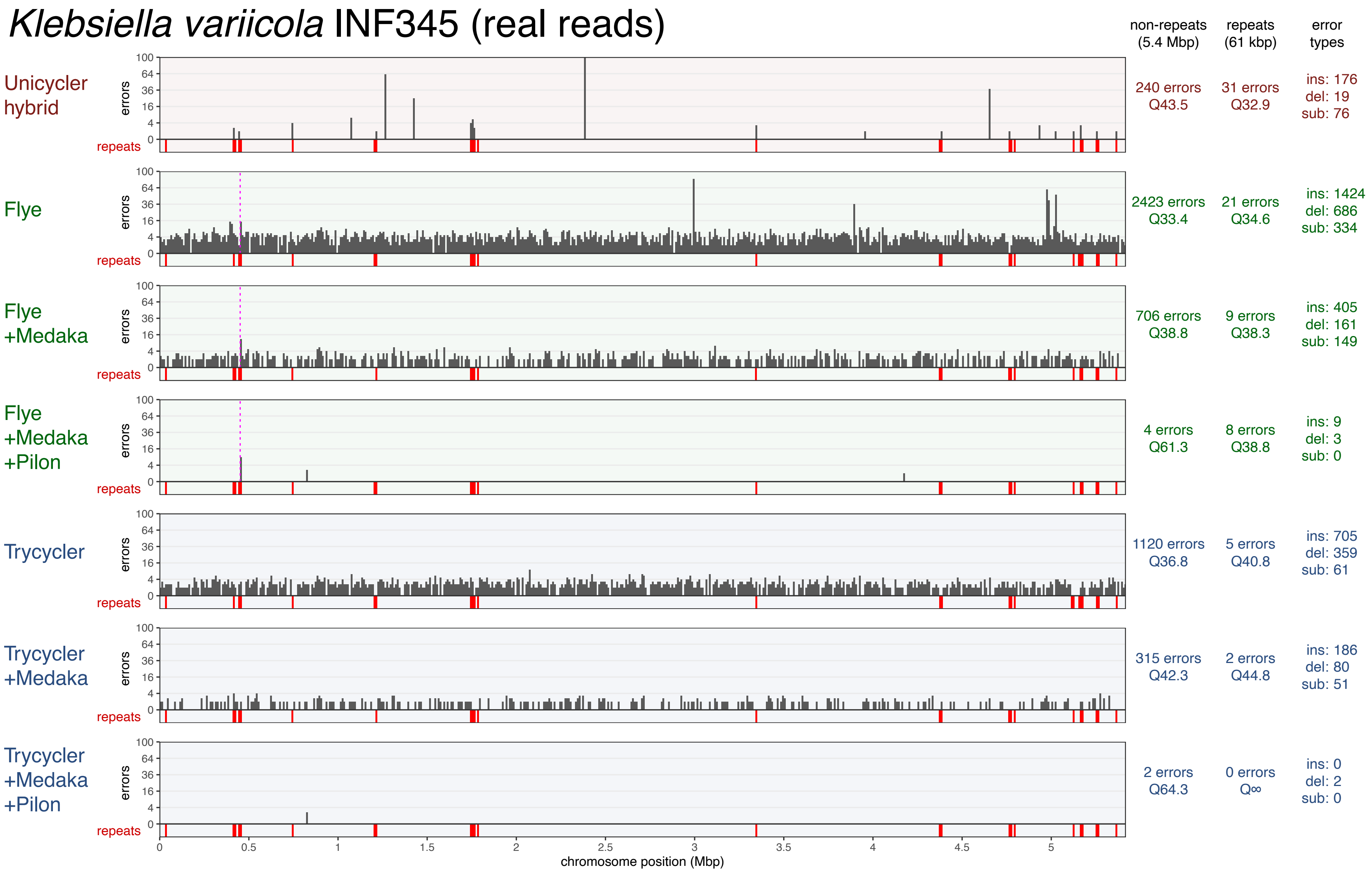

Fig. S4-p: error distributions (as defined by alignment between two independent assemblies) for the real-read assemblies of the *Klebsiella variicola* chromosome. Repetitive regions of the genome are shown as red blocks at the bottom of each plot. For Flye assemblies, the contig start/end positions (where the sequences began before they were rotated to be consistent with the reference) are shown by the dashed lines.

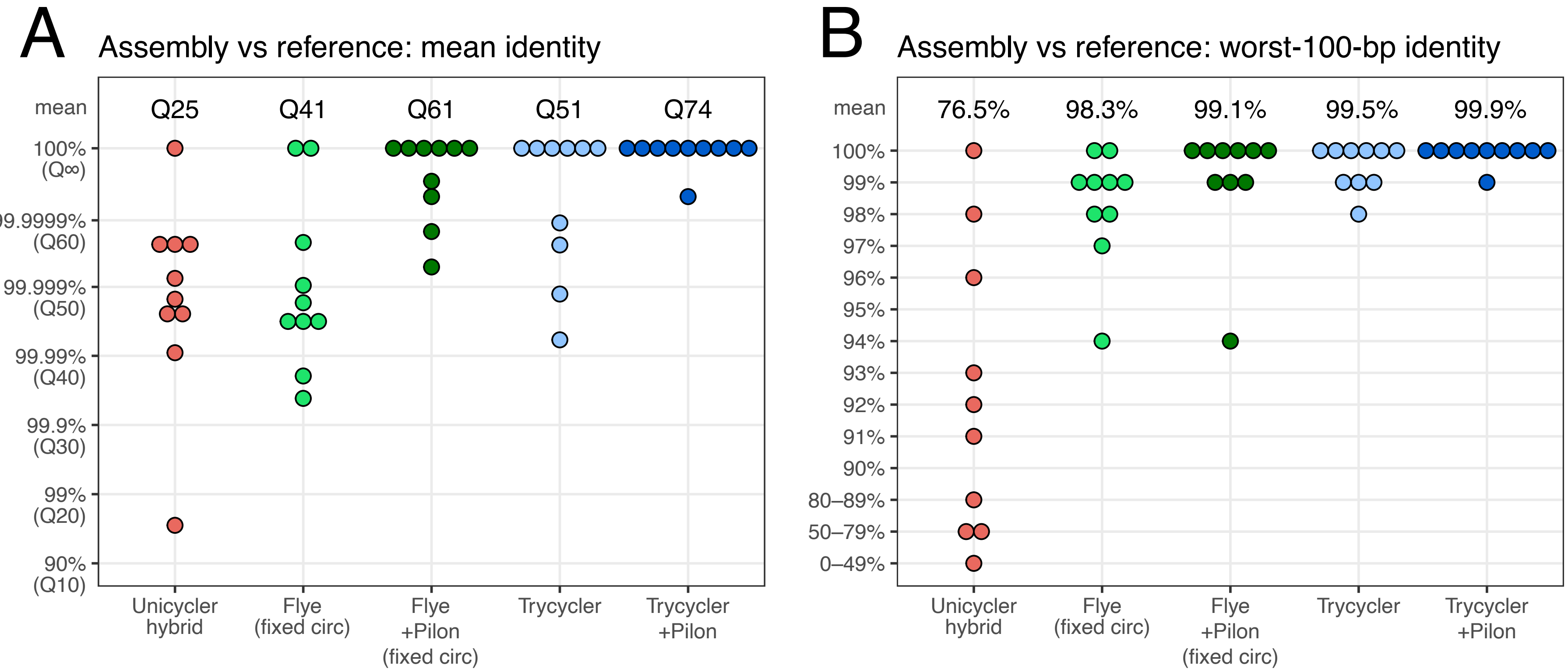

Fig. S5: results for the simulated read tests. This figure contains the same analyses as are shown in Figure 2, but the Flye assemblies have had their circularisation manually repaired.

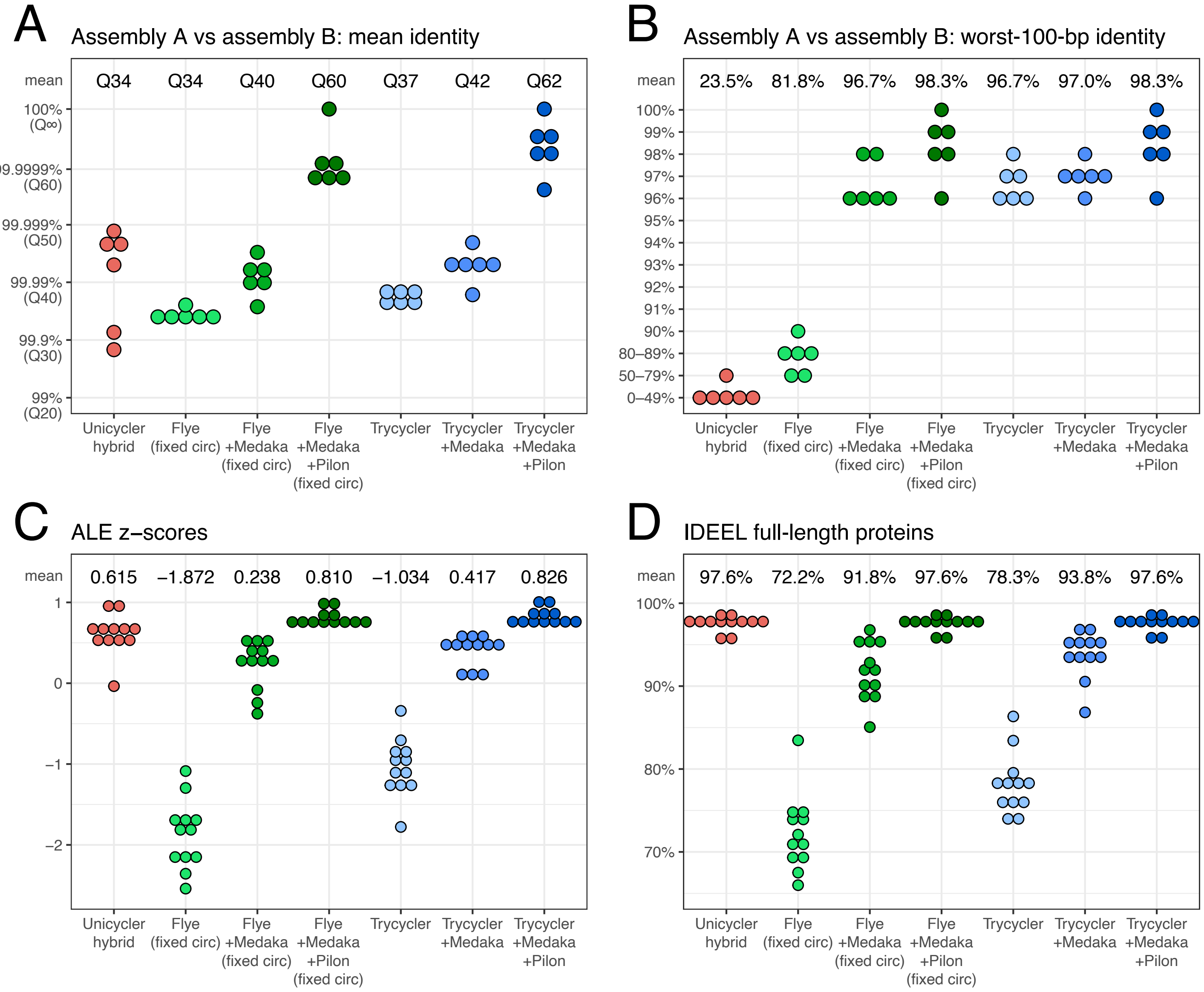

Fig. S6: results for the real read tests. This figure contains the same analyses as are shown in Figure 3, but the Flye assemblies have had their circularisation manually repaired.

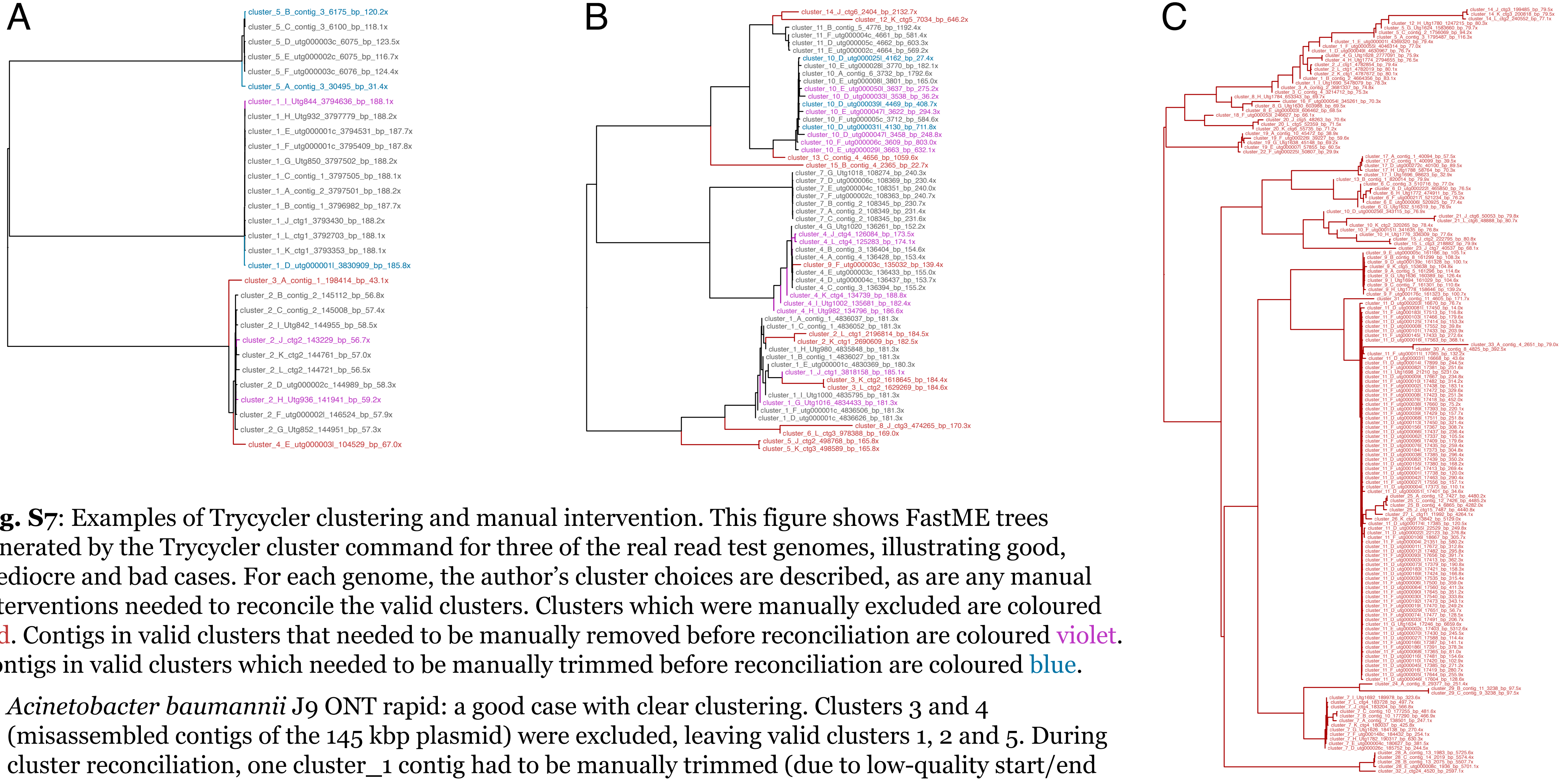

Fig. S7: Examples of Trycycler clustering and manual intervention. This figure shows FastME trees generated by the Trycycler cluster command for three of the real read test genomes, illustrating good, mediocre and bad cases. For each genome, the author's cluster choices are described, as are any manual interventions needed to reconcile the valid clusters. Clusters which were manually excluded are coloured red. Contigs in valid clusters that needed to be manually removed before reconciliation are coloured violet. Contigs in valid clusters which needed to be manually trimmed before reconciliation are coloured blue.

A. *Acinetobacter baumannii* J9 ONT rapid: a good case with clear clustering. Clusters 3 and 4 (misassembled contigs of the 145 kbp plasmid) were excluded, leaving valid clusters 1, 2 and 5. During cluster reconciliation, one cluster_1 contig had to be manually trimmed (due to low-quality start/end sequence), one cluster_1 contig had to be manually removed (due to poor pairwise alignment), two cluster_2 contigs had to be manually removed (due to being incomplete) and two cluster_5 contigs had to be manually trimmed (due to excessive length).

B. *Enterobacter kobei* MSB1_1B ONT rapid: a mediocre case with more complex clusters. Clusters 2, 3, 5, 6, 8 (misassembled contigs of the chromosome), 9 (misassembled contig of the 136 kbp plasmid), 12, 13, 14, and 15 (misassembled contigs of the small plasmids) were excluded, leaving valid clusters 1, 4, 7, 10 and 11. During cluster reconciliation, two cluster_1 contigs had to be manually removed (due to being incomplete or poor pairwise alignment), five cluster_4 contigs had to be manually removed (due to being incomplete), three cluster_10 contigs had to be manually trimmed (due to excessive length) and six cluster_10 contigs had to be manually removed (due to being incomplete or unable to circularise).

C. *Serratia marcescens* 17-147-1671 ONT rapid: a bad case where valid clusters were unclear. Insufficient read length and genome heterogeneity both contributed to the poor results. Without good clusters, it was not possible to proceed with Trycycler assembly.
