# Supplementary material for: Trycycler: consensus long-read assemblies for bacterial genomes
Source: Genome Biol. 2021 Sep 14;22:266. doi: 10.1186/s13059-021-02483-z (PMC8442456; doi:10.1186/s13059-021-02483-z)
Supplement: Supplementary file 2 — Additional file 2. Supplementary tables for the simulated-read tests. [file 13059_2021_2483_MOESM2_ESM.pdf]

The **References** worksheet contains the list of NCBI reference genomes, sorted number of genomes per species. References from the top 10 species are highlighted and were used as the genomes in the simulated read tests.

The **Genomes** worksheet contains information about the reference genomes that were used to generate the reads and as the ground truth against which assemblies were compared.

The **Read simulation** worksheet contains descriptions of the parameters used to generate Nanopore reads (made by Badread) and Illumina reads (made by ART).

The **Reads** worksheet contains summaries of the input read sets for each of the ten genomes. This includes both simulated Nanopore reads (made by Badread) and simulated Illumina reads (made by ART).

The **Assemblies** worksheet contains basic descriptions of each of the assemblies for each genome and computational stats.

The **Flye circularisation** worksheet shows the circularisation error in Flye assemblies (missing or duplicated bases at the start/end of contigs) which were manually fixed for the 'Flye (fixed circ)' assemblies.

The **Comparisons** worksheet contains chromosome identity values for each of the assemblies. These were based on an assembly-to-reference alignment of the chromosomal contig for each assembly. For Flye, values are given for both unaltered Flye assemblies ('Flye') and Flye assemblies where circularisation issue have been manually fixed ('Flye (fixed circ)').

The **Repeat identities** worksheet contains the sequence identities of all assemblies for both repeat and non-repeat regions of the genome. Identities are expressed both as percentages and a Q-scores.

References

#Species/genus	Genomes	Organism name	RefSeq accession	Chromosome RefSeq	Chromosome GenBank
Escherichia coli	15316	Escherichia coli str. K-12 substr. MG1655	GCF_000005845.2	NC_000913.3	U00096.3
Escherichia coli	15316	Escherichia coli O157:H7 str. Sakai		NC_002695.2	BA000007.3
Escherichia coli	15316	Escherichia coli IAI39		NC_011750.1	CU928164.2
Escherichia coli	15316	Escherichia coli UMN026		NC_011751.1	CU928163.2
Escherichia coli	15316	Escherichia coli O83:H1 str. NRG 857C		NC_017634.1	CP001855.1
Escherichia coli	15316	Escherichia coli O104:H4 str. 2011C-3493		NC_018658.1	CP003289.1
Salmonella enterica	13751	Salmonella enterica subsp. enterica serovar Typhimurium str. LT2	GCF_000006945.2	NC_003197.2	AE006468.2
Salmonella enterica	13751	Salmonella enterica subsp. enterica serovar Typhi str. CT18		NC_003198.1	AL513382.1
Staphylococcus aureus	10287	Staphylococcus aureus subsp. aureus NCTC 8325	GCF_000013425.1	NC_007795.1	CP000253.1
Streptococcus pneumoniae	8448	Streptococcus pneumoniae R6	GCF_000007045.1	NC_003098.1	AE007317.1
Klebsiella pneumoniae	6815	Klebsiella pneumoniae subsp. pneumoniae HS11286	GCF_000240185.1	NC_016845.1	CP003200.1
Mycobacterium tuberculosis	6456	Mycobacterium tuberculosis H37Rv	GCF_000195955.2	NC_000962.3	AL123456.3
Mycobacterium tuberculosis	6456	Mycobacterium tuberculosis variant bovis AF2122/97		NC_002945.4	LT708304.1
Pseudomonas aeruginosa	4107	Pseudomonas aeruginosa PAO1	GCF_000006765.1	NC_002516.2	AE004091.2
Listeria monocytogenes	3784	Listeria monocytogenes EGD-e	GCF_000196035.1	NC_003210.1	AL591824.1
Neisseria meningitidis	1938	Neisseria meningitidis MC58	GCF_000008805.1	NC_003112.2	AE002098.2
Campylobacter jejuni	1753	Campylobacter jejuni subsp. jejuni NCTC 11168 = ATCC 700819	GCF_000009085.1	NC_002163.1	AL111168.1
Mycobacteroides abscessus	1614	Mycobacteroides abscessus ATCC 19977	GCF_000069185.1	NC_010397.1	CU458896.1
Burkholderia pseudomallei	1533	Burkholderia pseudomallei K96243	GCF_000011545.1	NC_006350.1,NC_006351.1	BX571965.1,BX571966.1
Clostridioides difficile	1445	Clostridioides difficile 630	GCF_000009205.2	NC_009089.1	AM180355.1
Enterococcus faecium	1344	Enterococcus faecium DO	GCF_000174395.2	NC_017960.1	CP003583.1
Helicobacter pylori	1338	Helicobacter pylori 26695	GCF_000008525.1	NC_000915.1	AE000511.1
Streptococcus suis	1280	Streptococcus suis BM407	GCF_000026745.1	NC_012926.1	FM252032.1
Streptococcus agalactiae	1110	Streptococcus agalactiae 2603V/R	GCA_000007265.1	NC_004116.1	AE009948.1
Bacillus cereus	1016	Bacillus cereus ATCC 14579	GCF_000007825.1	NC_004722.1	AE016877.1
Vibrio cholerae	1002	Vibrio cholerae O1 biovar El Tor str. N16961	GCF_000006745.1	NC_002505.1,NC_002506.1	AE003852.1,AE003853.1
Enterococcus faecalis	880	Enterococcus faecalis V583	GCF_000007785.1	NC_004668.1	AE016830.1
Vibrio parahaemolyticus	872	Vibrio parahaemolyticus RIMD 2210633		NC_004605.1,NC_004603.1	BA000032.2,BA000031.2
Bordetella pertussis	765	Bordetella pertussis Tohama I		NC_002929.2	BX470248.1
Haemophilus influenzae	703	Haemophilus influenzae Rd KW20		NC_000907.1	L42023.1
Legionella pneumophila	638	Legionella pneumophila subsp. pneumophila str. Philadelphia 1		NC_002942.5	AE017354.1
Neisseria gonorrhoeae	573	Neisseria gonorrhoeae FA 1090		NC_002946.2	AE004969.1
Staphylococcus epidermidis	553	Staphylococcus epidermidis ATCC 12228		NC_004461.1	AE015929.1
Shigella flexneri	540	Shigella flexneri 2a str. 301		NC_004337.2	AE005674.2
Enterobacter cloacae	512	Enterobacter cloacae subsp. cloacae ATCC 13047		NC_014121.1	CP001918.1
Bacillus thuringiensis	499	[Bacillus thuringiensis] serovar konkukian str. 97-27		NC_005957.1	AE017355.1
Streptococcus pyogenes	489	Streptococcus pyogenes M1 GAS		NC_002737.2	AE004092.2
Pseudomonas syringae	386	Pseudomonas syringae pv. syringae B728a		NC_007005.1	CP000075.1
Yersinia pestis	378	Yersinia pestis CO92		NC_003143.1	AL590842.1
Lactobacillus plantarum	345	Lactobacillus plantarum WCFS1		NC_004567.2	AL935263.2
Leptospira interrogans	297	Leptospira interrogans serovar Lai str. 56601		NC_004343.2,NC_004342.2	AE010301.2,AE010300.2
Bacillus subtilis	252	Bacillus subtilis subsp. subtilis str. 168		NC_000964.3	AL009126.3
Clostridium botulinum	242	Clostridium botulinum A str. ATCC 3502		NC_009495.1	AM412317.1
Clostridium botulinum	242	Clostridium botulinum A str. Hall		NC_009698.1	CP000727.1
Francisella tularensis	235	Francisella tularensis subsp. tularensis SCHU S4		NC_006570.2	AJ749949.2
Bacillus anthracis	233	Bacillus anthracis str. Ames		NC_003997.3	AE016879.1
Bacillus anthracis	233	Bacillus anthracis str. Sterne		NC_005945.1	AE017225.1
Klebsiella aerogenes	210	Klebsiella aerogenes KCTC 2190		NC_015663.1	CP002824.1
Sinorhizobium meliloti	204	Sinorhizobium meliloti 1021		NC_003047.1	AL591688.1
Acinetobacter pittii	202	Acinetobacter pittii PHEA-2		NC_016603.1	CP002177.1
Streptococcus mutans	194	Streptococcus mutans UA159		NC_004350.2	AE014133.2
Bifidobacterium longum	189	Bifidobacterium longum NCC2705		NC_004307.2	AE014295.3
Yersinia enterocolitica	181	Yersinia enterocolitica subsp. enterocolitica 8081		NC_008800.1	AM286415.1
Lactococcus lactis	180	Lactococcus lactis subsp. lactis Il1403		NC_002662.1	AE005176.1
Chlamydia trachomatis	176	Chlamydia trachomatis D/UW-3/CX		NC_000117.1	AE001273.1
Chlamydia trachomatis	176	Chlamydia trachomatis 434/Bu		NC_010287.1	AM884176.1
Bacteroides fragilis	160	Bacteroides fragilis YCH46		NC_006347.1	AP006841.1
Lactobacillus paracasei	157	Lactobacillus paracasei ATCC 334		NC_008526.1	CP000423.1
Pseudomonas putida	118	Pseudomonas putida KT2440		NC_002947.4	AE015451.2
Borreliella burgdorferi	111	Borreliella burgdorferi B31		NC_001318.1	AE000783.1
Gardnerella vaginalis	104	Gardnerella vaginalis ATCC 14019		NC_014644.1	CP002104.1
Xanthomonas campestris	94	Xanthomonas campestris pv. campestris str. ATCC 33913		NC_003902.1	AE008922.1
Streptococcus mitis	91	Streptococcus mitis B6		NC_013853.1	FN568063.1
Bordetella bronchiseptica	91	Bordetella bronchiseptica 253		NC_019382.1	HE965806.1
Mycoplasma pneumoniae	89	Mycoplasma pneumoniae M129		NC_000912.1	U00089.2
Lactobacillus salivarius	84	Lactobacillus salivarius UCC118		NC_007929.1	CP000233.1
Burkholderia mallei	83	Burkholderia mallei ATCC 23344		NC_006349.2,NC_006348.1	CP000011.2,CP000010.1
Aeromonas hydrophila	73	Aeromonas hydrophila subsp. hydrophila ATCC 7966		NC_008570.1	CP000462.1
Flavobacterium psychrophilum	73	Flavobacterium psychrophilum JIP02/86		NC_009613.3	AM398681.2
Shigella dysenteriae	72	Shigella dysenteriae Sd197		NC_007606.1	CP000034.1
Bifidobacterium bifidum	69	Bifidobacterium bifidum PRL2010		NC_014638.1	CP001840.1
Coxiella burnetii	68	Coxiella burnetii RSA 493		NC_002971.4	AE016828.3
Fusobacterium nucleatum	68	Fusobacterium nucleatum subsp. nucleatum ATCC 25586		NC_003454.1	AE009951.2
Pseudomonas syringae group ge	68	Pseudomonas syringae pv. tomato str. DC3000		NC_004578.1	AE016853.1
Streptococcus sanguinis	62	Streptococcus sanguinis SK36		NC_009009.1	CP000387.1
Prochlorococcus marinus	48	Prochlorococcus marinus subsp. marinus str. CCMP1375		NC_005042.1	AE017126.1
Sinorhizobium medicae	42	Sinorhizobium medicae WSM419		NC_009636.1	CP000738.1
Corynebacterium glutamicum	39	Corynebacterium glutamicum ATCC 13032		NC_003450.3	BA000036.3
Lactobacillus acidophilus	39	Lactobacillus acidophilus NCFM		NC_006814.3	CP000033.3
Bacteroides thetaiotaomicron	35	Bacteroides thetaiotaomicron VPI-5482		NC_004663.1	AE015928.1
Aliivibrio fischeri	34	Aliivibrio fischeri ES114		NC_006840.2,NC_006841.2	CP000020.2,CP000021.2
Buchnera aphidicola	30	Buchnera aphidicola str. APS (Acyrthosiphon pisum)		NC_002528.1	BA000003.2
Mycoplasma mycoides	23	Mycoplasma mycoides subsp. mycoides SC str. PG1		NC_005364.2	BX293980.2
Rhodobacter sphaeroides	21	Rhodobacter sphaeroides 2.4.1		NC_007494.2,NC_007493.2	CP000144.2,CP000143.2
Bordetella parapertussis	21	Bordetella parapertussis Bpp5		NC_018828.1	HE965803.1
Sinorhizobium fredii	18	Sinorhizobium fredii NGR234		NC_012587.1	CP001389.1
Treponema denticola	17	Treponema denticola ATCC 35405		NC_002967.9	AE017226.1
Chlamydia pneumoniae	13	Chlamydia pneumoniae CWL029		NC_000922.1	AE001363.1
Rickettsia prowazekii	13	Rickettsia prowazekii str. Madrid E		NC_000963.1	AJ235269.1
Mesoplasma florum	12	Mesoplasma florum L1		NC_006055.1	AE017263.1
Moorella thermoacetica	12	Moorella thermoacetica ATCC 39073		NC_007644.1	CP000232.1
Caulobacter vibrioides	11	Caulobacter vibrioides CB15		NC_002696.2	AE005673.1
Salinibacter ruber	11	Salinibacter ruber DSM 13855		NC_007677.1	CP000159.1
Caulobacter vibrioides	11	Caulobacter vibrioides NA1000		NC_011916.1	CP001340.1
Clostridium acetobutylicum	10	Clostridium acetobutylicum ATCC 824		NC_003030.1	AE001437.1
Thermus thermophilus	10	Thermus thermophilus HB8		NC_006461.1	AP008226.1
Bradyrhizobium diazoefficiens	9	Bradyrhizobium diazoefficiens USDA 110		NC_004463.1	BA000040.2
Mycolicibacterium smegmatis	9	Mycolicibacterium smegmatis MC2 155		NC_008596.1	CP000480.1
Amycolatopsis mediterranei	7	Amycolatopsis mediterranei U32		NC_014318.1	CP002000.1
Thermotoga maritima	6	Thermotoga maritima MSB8		NC_000853.1	AE000512.1
Mycobacterium leprae	6	Mycobacterium leprae TN		NC_002677.1	AL450380.1
Geobacter sulfurreducens	6	Geobacter sulfurreducens PCA		NC_002939.5	AE017180.2
Agrobacterium fabrum	6	Agrobacterium fabrum str. C58		NC_003063.2,NC_003062.2	AE007870.2,AE007869.2
Rhodopirellula baltica	6	Rhodopirellula baltica SH 1		NC_005027.1	BX119912.1
Mesorhizobium ciceri	6	Mesorhizobium ciceri biovar biserrulae WSM1271		NC_014923.1	CP002447.1
Deinococcus radiodurans	5	Deinococcus radiodurans R1		NC_001263.1,NC_001264.1	AE000513.1,AE001825.1
Ketogulonicigenium vulgare	5	Ketogulonicigenium vulgare WSH-001		NC_017384.1	CP002018.1
Desulfovibrio vulgaris	4	Desulfovibrio vulgaris str. Hildenborough		NC_002937.3	AE017285.1
Rhodospirillum rubrum	4	Rhodospirillum rubrum ATCC 11170		NC_007643.1	CP000230.1
Chloroflexus aurantiacus	3	Chloroflexus aurantiacus J-10-fl		NC_010175.1	CP000909.1
Brachybacterium faecium	3	Brachybacterium faecium DSM 4810		NC_013172.1	CP001643.1
Thermosynechococcus elongatus	2	Thermosynechococcus elongatus BP-1		NC_004113.1	BA000039.2
Shewanella oneidensis	2	Shewanella oneidensis MR-1		NC_004347.2	AE014299.2
Dictyoglomus turgidum	2	Dictyoglomus turgidum DSM 6724		NC_011661.1	CP001251.1
Aquifex aeolicus	1	Aquifex aeolicus VF5		NC_000918.1	AE000657.1
Chlorobaculum tepidum	1	Chlorobaculum tepidum TLS		NC_002932.3	AE006470.1
Streptomyces coelicolor	1	Streptomyces coelicolor A3(2)		NC_003888.3	AL645882.2
Gloeobacter violaceus	1	Gloeobacter violaceus PCC 7421		NC_005125.1	BA000045.2
Thermodesulfovibrio yellowstoni	1	Thermodesulfovibrio yellowstonii DSM 11347		NC_011296.1	CP001147.1
Thermanaerovibrio acidaminovo	1	Thermanaerovibrio acidaminovorans DSM 6589		NC_013522.1	CP001818.1

Genomes

Species	RefSeq accession	Genome size	Replicons	Longest repeat	
				From Unicycler	From MUMmer
Campylobacter jejuni	GCF_000009085.1	1,641,481	1	21,305	6,101
Escherichia coli	GCF_000005845.2	4,641,652	1	6,772	5,463
Klebsiella pneumoniae	GCF_000240185.1	5,682,322	7	7,036	9,161
Listeria monocytogenes	GCF_000196035.1	2,944,528	1	14,849	5,934
Mycobacterium tuberculosis	GCF_000195955.2	4,411,532	1	10,195	2,441
Neisseria meningitidis	GCF_000008805.1	2,272,360	1	12,532	32,194
Pseudomonas aeruginosa	GCF_000006765.1	6,264,404	1	9,951	6,329
Salmonella enterica	GCF_000006945.2	4,951,383	2	11,657	11,668
Staphylococcus aureus	GCF_000013425.1	2,821,361	1	9,381	5,404
Streptococcus pneumoniae	GCF_000007045.1	2,038,615	1	8,843	6,080

Read simulation

Sample	Badread parameters									ART parameters				
	quantity	length	identity	start_adapter_seq	end_adapter_seq	junk	random	chimeras	glitches	seqSys	len	fcov	mflen	sdev
Campylobacter jejuni	200x	20788,06361	94,097,2	26	15	0	0	1	7083,40,25	MSv1	250	90	340	53
Escherichia coli	280x	26103,08384	93,095,5	2	40	0	0	1	5766,28,08	HS25	150	110	420	21
Klebsiella pneumoniae	210x	14688,12402	91,095,5	2	44	0	0	2	3422,44,04	HSXt	150	40	500	122
Listeria monocytogenes	150x	30814,24000	96,100,3	9	16	0	0	1	8367,28,14	MSv3	250	130	300	30
Mycobacterium tuberculosis	270x	25285,19637	93,096,4	2	19	0	0	3	7377,10,28	HS25	125	70	460	31
Neisseria meningitidis	250x	37314,27176	96,098,5	27	14	0	0	1	6710,44,32	HS20	100	120	540	45
Pseudomonas aeruginosa	260x	35001,18994	93,100,5	21	2	0	0	2	6726,25,27	HS25	125	80	660	51
Salmonella enterica	120x	35097,26894	91,097,2	43	12	0	0	2	8267,31,28	HS20	100	50	580	61
Staphylococcus aureus	190x	36588,29376	93,095,4	23	14	0	0	0	4138,03,07	HS25	150	60	380	81
Streptococcus pneumoniae	230x	13506,05927	93,096,3	43	2	0	0	2	7045,14,11	HSXn	150	100	620	117

Reads

Sample	Nanopore reads						Illumina reads					
	All			After Filtlong			All		After fastp		Insert size	
	Read count	Total size	N50 length	Read count	Total size	N50 length	Read count	Total size	Read count	Total size	Min	Max
Campylobacter jejuni	15,899	328,306,798	21,857	14,928	311,892,601	22,060	590,932	147,733,000	590,932	147,733,000	255	465
Escherichia coli	49,890	1,299,671,812	27,746	47,015	1,234,698,132	27,919	3,403,874	510,581,100	3,403,874	510,530,164	371	469
Klebsiella pneumoniae	82,543	1,193,314,755	21,903	63,165	1,133,657,145	22,808	1,576,992	236,548,800	1,576,992	236,536,750	224	784
Listeria monocytogenes	14,298	441,712,781	44,029	12,144	419,627,702	45,127	1,531,154	382,788,500	1,531,154	382,788,500	253	370
Mycobacterium tuberculosis	46,881	1,191,127,196	35,763	39,001	1,131,573,946	36,767	2,470,454	308,806,750	2,470,454	308,783,950	387	534
Neisseria meningitidis	15,314	568,095,071	49,909	13,266	539,720,684	50,644	2,726,832	272,683,200	2,726,832	272,495,564	433	653
Pseudomonas aeruginosa	46,435	1,628,785,042	42,003	42,376	1,547,378,832	42,658	4,009,216	501,152,000	4,009,216	501,141,756	541	779
Salmonella enterica	16,818	594,179,503	49,693	14,866	564,494,157	50,514	2,494,476	249,447,600	2,494,476	249,440,222	437	723
Staphylococcus aureus	14,770	536,073,199	52,657	12,392	509,272,441	53,772	1,128,424	169,263,600	1,128,424	169,220,628	197	570
Streptococcus pneumoniae	34,436	468,903,406	15,366	31,175	445,462,552	15,661	1,359,070	203,860,500	1,359,070	203,832,422	346	893

Assemblies

Sample	Unicycler hybrid					Miniasm					Raven					Flye					Trycycler				
	Assembly size	Contigs	Complete chromosome	Time (h:m:s)	RAM (kB)	Assembly size	Contigs	Complete chromosome	Time (h:m:s)	RAM (kB)	Assembly size	Contigs	Complete chromosome	Time (h:m:s)	RAM (kB)	Assembly size	Contigs	Complete chromosome	Time (h:m:s)	RAM (kB)	Assembly size	Contigs	Complete chromosome	Time (h:m:s)	RAM (kB)
Campylobacter jejuni	1,641,472	1	yes	0:57:03	12,170,432	1,641,573	1	yes	0:04:25	6,210,684	1,641,386	1	yes	0:01:20	2,865,080	1,641,424	1	yes	0:27:42	9,601,036	1,641,477	1	yes	0:39:43	8,385,200
Escherichia coli	4,641,651	1	yes	2:33:19	21,301,536	4,642,148	1	yes	0:19:55	11,025,428	4,641,776	1	yes	0:06:01	11,361,088	4,641,573	1	yes	0:47:38	14,448,860	4,641,647	1	yes	1:14:21	11,458,888
Klebsiella pneumoniae	5,682,317	7	yes	2:15:26	22,701,332	5,675,071	4	yes	0:17:14	11,872,580	5,673,918	4	yes	0:05:55	11,073,836	5,682,179	5	yes	0:28:50	11,639,508	5,673,839	4	yes	1:31:58	10,309,436
Listeria monocytogenes	2,944,529	1	yes	1:47:10	7,639,920	2,944,620	1	yes	0:05:46	13,679,532	2,942,987	1	yes	0:01:43	4,015,320	2,944,529	1	yes	0:33:17	21,489,260	2,944,528	1	yes	0:59:10	20,276,076
Mycobacterium tuberculosis	4,411,523	1	yes	4:01:05	44,198,720	4,411,853	1	yes	0:18:00	15,271,984	4,411,552	1	yes	0:05:42	10,977,824	4,411,490	1	yes	0:47:51	22,155,296	4,411,532	1	yes	1:12:17	17,770,864
Neisseria meningitidis	2,240,343	2	almost	4:13:26	24,426,200	2,272,506	1	yes	0:09:57	25,029,344	2,272,363	1	yes	0:02:50	5,167,476	2,272,351	1	yes	1:41:49	38,550,464	2,272,360	1	yes	1:20:51	23,669,660
Pseudomonas aeruginosa	6,264,411	1	yes	6:46:48	21,115,984	6,265,024	1	yes	0:25:42	19,329,416	6,264,482	1	yes	0:08:05	15,202,356	6,264,389	1	yes	1:07:01	25,104,308	6,264,404	1	yes	1:37:02	23,946,104
Salmonella enterica	4,951,381	2	yes	2:20:02	21,986,216	4,951,515	2	yes	0:07:18	7,551,480	4,951,147	2	yes	0:03:00	5,477,756	4,949,345	2	yes	0:23:51	18,126,620	4,951,093	2	yes	1:18:29	20,650,204
Staphylococcus aureus	2,821,354	1	yes	1:56:27	16,762,932	2,821,514	1	yes	0:07:04	12,853,784	2,821,323	1	yes	0:02:42	4,893,240	2,821,283	1	yes	0:27:30	21,566,232	2,821,361	1	yes	1:04:58	26,676,764
Streptococcus pneumoniae	2,038,615	1	yes	2:12:50	11,581,680	2,038,746	1	yes	0:06:19	6,947,452	2,038,540	1	yes	0:01:48	4,248,164	2,038,534	1	yes	0:22:53	7,140,940	2,038,615	1	yes	0:38:18	5,634,940

Flye circularisation

Assembly	Circ error size	Extra bases	Missing bases
Campylobacter jejuni	3	ATG	
Escherichia coli	0		
Klebsiella pneumoniae	-7		TTTGATG
Listeria monocytogenes	1	A	
Mycobacterium tuberculosis	0		
Neisseria meningitidis	-9		AGACGGCAT
Pseudomonas aeruginosa	-1		C
Salmonella enterica	-6		GGAGTT
Staphylococcus aureus	-9		ATGAACATT
Streptococcus pneumoniae	2	AA	

Mean error size: 3.8

Comparisons

Sample	Unicycler		Miniasm		Miniasm+Pilon		Raven		Raven+Pilon		Flye		Flye+Pilon		Flye (fixed circ)		Flye (fixed circ) +Pilon		Trycycler		Trycycler+Pilon	
	Mean identity	Worst 100 bp identity	Mean identity	Worst 100 bp identity	Mean identity	Worst 100 bp identity	Mean identity	Worst 100 bp identity	Mean identity	Worst 100 bp identity	Mean identity	Worst 100 bp identity	Mean identity	Worst 100 bp identity	Mean identity	Worst 100 bp identity	Mean identity	Worst 100 bp identity	Mean identity	Worst 100 bp identity	Mean identity	Worst 100 bp identity
Campylobacter jejuni	99.99098%	54%	99.98093%	90%	99.99994%	99%	99.98672%	26%	99.99525%	26%	99.99616%	97%	99.99982%	97%	99.99634%	98%	100.00000%	100%	99.99976%	99%	100.00000%	100%
Escherichia coli	99.99849%	92%	99.98126%	91%	99.99994%	98%	99.98759%	88%	99.99925%	88%	99.99830%	98%	99.99998%	99%	99.99830%	98%	99.99998%	99%	99.99989%	99%	100.00000%	100%
Klebsiella pneumoniae	99.99981%	98%	99.96261%	88%	99.99953%	89%	99.97421%	21%	99.99745%	21%	99.98030%	93%	99.99972%	93%	99.98043%	97%	99.99985%	99%	99.99873%	99%	100.00000%	100%
Listeria monocytogenes	99.99925%	96%	99.99463%	91%	100.00000%	100%	99.94267%	0%	99.94573%	0%	99.99997%	99%	99.99997%	99%	100.00000%	100%	100.00000%	100%	100.00000%	100%	100.00000%	100%
Mycobacterium tuberculosis	99.99971%	91%	99.98629%	95%	99.99991%	97%	99.99218%	84%	99.99959%	84%	99.99905%	99%	100.00000%	100%	99.99905%	99%	100.00000%	100%	100.00000%	100%	100.00000%	100%
Neisseria meningitidis	97.17851%	0%	99.98944%	83%	99.99780%	83%	99.99529%	89%	99.99930%	89%	99.99960%	91%	99.99960%	91%	100.00000%	100%	100.00000%	100%	100.00000%	100%	100.00000%	100%
Pseudomonas aeruginosa	99.99978%	93%	99.98461%	90%	99.99994%	97%	99.99468%	61%	99.99938%	61%	99.99976%	99%	99.99998%	99%	99.99978%	99%	100.00000%	100%	100.00000%	100%	100.00000%	100%
Salmonella enterica	99.99714%	57%	99.97952%	93%	99.99979%	96%	99.98744%	26%	99.99802%	26%	99.95862%	93%	99.99936%	94%	99.95874%	94%	99.99949%	94%	99.99413%	98%	99.99996%	99%
Staphylococcus aureus	99.99787%	84%	99.98834%	96%	100.00000%	100%	99.99249%	57%	99.99848%	57%	99.99720%	91%	99.99965%	91%	99.99752%	99%	99.99996%	99%	100.00000%	100%	100.00000%	100%
Streptococcus pneumoniae	100.00000%	100%	99.98519%	92%	99.99990%	98%	99.99166%	56%	99.99784%	56%	99.99583%	98%	99.99990%	98%	99.99593%	99%	100.00000%	100%	100.00000%	100%	100.00000%	100%

Repeat identities

Sample	Unicycler A vs B				Flye A vs B				Flye +Pilon A vs B				Flye (fixed circ) A vs B				Flye (fixed circ) +Pilon A vs B				Trycycler A vs B				Trycycler +Pilon A vs B			
	Non-repeat identity	Non-repeat Qscore	Repeat identity	Repeat Qscore	Non-repeat identity	Non-repeat Qscore	Repeat identity	Repeat Qscore	Non-repeat identity	Non-repeat Qscore	Repeat identity	Repeat Qscore	Non-repeat identity	Non-repeat Qscore	Repeat identity	Repeat Qscore	Non-repeat identity	Non-repeat Qscore	Repeat identity	Repeat Qscore	Non-repeat identity	Non-repeat Qscore	Repeat identity	Repeat Qscore	Non-repeat identity	Non-repeat Qscore	Repeat identity	Repeat Qscore
Campylobacter jejuni	100.00000%	inf	99.60099%	24.0	99.99628%	44.3	99.99008%	40.0	99.99981%	57.3	100.00000%	inf	99.99646%	44.5	99.99008%	40.0	100.00000%	inf	100.00000%	inf	99.99988%	59.1	99.99339%	41.8	100.00000%	inf	100.00000%	inf
Escherichia coli	100.00000%	inf	99.93490%	31.9	99.99830%	47.7	99.99814%	47.3	100.00000%	inf	99.99907%	50.3	99.99830%	47.7	99.99814%	47.3	100.00000%	inf	99.99907%	50.3	99.99989%	59.6	100.00000%	inf	100.00000%	inf	100.00000%	inf
Klebsiella pneumoniae	99.99998%	67.2	99.98972%	39.9	99.98035%	37.1	99.97715%	36.4	99.99998%	67.2	99.98400%	38.0	99.98035%	37.1	99.98515%	38.3	99.99998%	67.2	99.99200%	41.0	99.99870%	48.9	100.00000%	inf	100.00000%	inf	100.00000%	inf
Listeria monocytogenes	100.00000%	inf	99.94078%	32.3	100.00000%	inf	99.99731%	45.7	100.00000%	inf	99.99731%	45.7	100.00000%	inf	100.00000%	inf	100.00000%	inf	100.00000%	inf	100.00000%	inf	100.00000%	inf	100.00000%	inf	100.00000%	inf
Mycobacterium tuberculosis	99.99977%	56.4	99.99440%	42.5	99.99904%	50.2	100.00000%	inf	100.00000%	inf	100.00000%	inf	99.99904%	50.2	100.00000%	inf	100.00000%	inf	100.00000%	inf	100.00000%	inf	100.00000%	inf	100.00000%	inf	100.00000%	inf
Neisseria meningitidis	99.46904%	22.7	73.47983%	5.8	100.00000%	inf	99.99541%	43.4	100.00000%	inf	99.99541%	43.4	100.00000%	inf	100.00000%	inf	100.00000%	inf	100.00000%	inf	100.00000%	inf	100.00000%	inf	100.00000%	inf	100.00000%	inf
Pseudomonas aeruginosa	99.99989%	59.5	99.99264%	41.3	99.99979%	56.8	99.99790%	46.8	100.00000%	inf	99.99895%	49.8	99.99979%	56.8	99.99895%	49.8	100.00000%	inf	100.00000%	inf	100.00000%	inf	100.00000%	inf	100.00000%	inf	100.00000%	inf
Salmonella enterica	99.99947%	52.8	99.88451%	29.4	99.95896%	33.9	99.94211%	32.4	99.99987%	59.0	99.97461%	36.0	99.95896%	33.9	99.94820%	32.9	99.99987%	59.0	99.98070%	37.1	99.99416%	42.3	99.99289%	41.5	100.00000%	inf	99.99797%	46.9
Staphylococcus aureus	100.00000%	inf	99.86436%	28.7	99.99748%	46.0	99.97965%	36.9	99.99993%	61.4	99.98191%	37.4	99.99752%	46.0	99.99774%	46.5	99.99996%	64.4	100.00000%	inf	100.00000%	inf	100.00000%	inf	100.00000%	inf	100.00000%	inf
Streptococcus pneumoniae	100.00000%	inf	100.00000%	inf	99.99575%	43.7	100.00000%	inf	99.99990%	60.0	100.00000%	inf	99.99585%	43.8	100.00000%	inf	100.00000%	inf	100.00000%	inf	100.00000%	inf	100.00000%	inf	100.00000%	inf	100.00000%	inf
**Average (mean)**	**99.94091%**	**32.3**	**96.96468%**	**15.2**	**99.99224%**	**41.1**	**99.98642%**	**38.7**	**99.99996%**	**63.5**	**99.99236%**	**41.2**	**99.99227%**	**41.1**	**99.99092%**	**40.4**	**99.99998%**	**67.0**	**99.99686%**	**45.0**	**99.99918%**	**50.9**	**99.99848%**	**48.2**	**100.00000%**	**inf**	**99.99977%**	**56.5**
