# Supplementary material for: Trycycler: consensus long-read assemblies for bacterial genomes
Source: Genome Biol. 2021 Sep 14;22:266. doi: 10.1186/s13059-021-02483-z (PMC8442456; doi:10.1186/s13059-021-02483-z)
Supplement: Supplementary file 3 — Additional file 3. Supplementary tables for the real-read tests. [file 13059_2021_2483_MOESM3_ESM.pdf]

The **Genomes** worksheet contains information about the genomes that were used to generate the reads.

The **Reads** worksheet contains descriptions of the input read sets for each of the seven real genomes. This includes ligation-prep and rapid-prep Nanopore reads, as well as Illumina reads. Illumina reads were evenly split into two groups: group A was paired with ligation-prep Nanopore reads and group B was paired with rapid-prep Nanopore reads.

The **Assemblies** worksheet contains basic descriptions of each of the assemblies for each genome and computational stats. Some genome-level stats are also provided here, such as the longest repeat in each genome.

The **Flye circularisation** worksheet shows the circularisation error in Flye assemblies (missing or duplicated bases at the start/end of contigs) which were manually fixed for the 'Flye (fixed circ)' assemblies.

The **Comparisons** worksheet contains chromosome identity values for each of the assemblies. These were based on aligning the chromosomal contig for each read set A assembly to the chromosomal contig in the read set B assembly. For Flye, values are given for both unaltered Flye assemblies ('Flye') and Flye assemblies where circularisation issues have been manually fixed ('Flye (fixed circ)'). Note that not all assemblies for *Serratia marcescens* 17-147-1671 produced a complete chromosomal contig, and so this genome is excluded from this and subsequent worksheets.

The **ALE scores** worksheet contains overall ALE scores for each of the assemblies of the six analysed genomes. Note that this worksheet uses the unaltered Flye assemblies.

The **ALE scores (fixed circ)** contains the same data as the previous worksheet, except that it uses the Flye assemblies with manually-fixed circularisation.

The **ALE z-scores** worksheet contains the same data as the ALE scores worksheet, except that each score has been converted into a z-score (mean of 0 and stdev of 1) on a per-genome basis.

The **ALE z-scores (fixed circ)** worksheet contains the same data as the ALE scores (fixed circ) worksheet, except that each score has been converted into a z-score (mean of 0 and stdev of 1) on a per-genome basis.

The **Ideel results** worksheet contains the fraction of protein sequences in each assembly with an Ideel ratio of 95% or more. Note that this worksheet uses the unaltered Flye assemblies.

The **Ideel results (fixed circ)** contains the same data as the previous worksheet, except that it uses the Flye assemblies with manually-fixed circularisation.

The **Repeat identities** worksheet contains the sequence identities of all assemblies for both repeat and non-repeat regions of the genome. Identities are expressed both as percentages and a Q-scores.

The **Medaka order** worksheet contains results (same format as the Comparisons worksheet) for a few different orders of running Trycycler and Medaka.

Genomes

Sample	Genome size	Replicons	Longest repeat: From Unicycler	Longest repeat: From MUMmer
Acinetobacter baumannii J9	3,949,783	3	10,246	5,842
Citrobacter koseri MINF_9D	4,833,163	3	29,556	28,824
Enterobacter kobei MSB1_1B	5,093,569	6	14,947	12,473
Haemophilus M1C132_1	2,124,989	6	7,322	7,896
Klebsiella oxytoca MSB1_2C	5,995,634	5	10,787	5,622
Klebsiella variicola INF345	5,952,932	6	5,656	5,582
Serratia marcescens 17-147-1671	5,883,278	5	39,060	39,051

Reads

Sample	Read set A											
	Nanopore ligation reads						Illumina reads - group A					
	All			After Filtlong			All		After fastp		Insert size	
	Read count	Total size	N50 length	Read count	Total size	N50 length	Read count	Total size	Read count	Total size	Min	Max
Acinetobacter baumannii J9	85,484	1,070,356,827	21,638	60,765	1,016,840,396	22,623	3,115,226	454,925,397	3,073,586	448,907,913	157	712
Citrobacter koseri MINF_9D	139,899	1,009,600,962	18,781	79,387	959,121,239	19,909	2,284,300	332,880,816	2,247,899	327,687,679	159	721
Enterobacter kobei MSB1_1B	45,191	625,579,715	21,856	34,327	594,322,331	22,638	2,046,868	298,913,783	2,016,063	294,505,747	160	719
Haemophilus M1C132_1	220,304	1,342,021,782	16,260	127,998	1,274,921,933	17,502	1,983,498	290,238,947	1,953,677	285,707,813	160	758
Klebsiella oxytoca MSB1_2C	36,750	483,128,849	24,765	25,498	458,972,442	25,971	3,637,684	530,737,946	3,586,483	523,267,046	157	716
Klebsiella variicola INF345	167,862	1,452,069,004	25,814	80,509	1,379,466,058	27,115	1,666,578	242,275,969	1,640,587	238,549,905	156	704
Serratia marcescens 17-147-1671	139,058	1,091,695,992	20,731	81,121	1,037,111,473	22,098	1,585,894	232,121,118	1,556,040	227,792,294	158	734

Sample	Read set B											
	Nanopore rapid reads						Illumina reads - group B					
	All			After Filtlong			All		After fastp		Insert size	
	Read count	Total size	N50 length	Read count	Total size	N50 length	Read count	Total size	Read count	Total size	Min	Max
Acinetobacter baumannii J9	101,526	780,286,224	14,351	69,135	741,274,205	15,130	3,115,226	454,886,971	3,073,051	448,801,170	157	713
Citrobacter koseri MINF_9D	213,402	1,666,038,163	14,142	143,774	1,582,736,706	14,924	2,284,300	332,916,017	2,247,656	327,668,697	159	721
Enterobacter kobei MSB1_1B	128,031	987,293,009	15,407	80,886	937,930,249	16,234	2,046,866	298,849,729	2,016,193	294,466,039	160	717
Haemophilus M1C132_1	271,823	1,704,343,789	10,276	185,001	1,619,105,604	10,569	1,983,498	290,147,888	1,953,695	285,623,168	160	757
Klebsiella oxytoca MSB1_2C	105,226	808,961,843	14,406	69,978	768,514,620	15,181	3,637,682	530,767,101	3,587,102	523,390,295	157	716
Klebsiella variicola INF345	132,640	998,133,789	14,587	85,440	948,228,758	15,364	1,666,578	242,310,384	1,640,733	238,588,432	156	705
Serratia marcescens 17-147-1671	138,974	699,122,054	9,898	94,341	664,166,169	10,457	1,585,894	232,065,297	1,556,079	227,754,139	158	755

Assemblies

Sample	Unicycler hybrid A					Unicycler hybrid B				
	Assembly size	Contigs	Complete chromosome	Time (h:m:s)	RAM (kB)	Assembly size	Contigs	Complete chromosome	Time (h:m:s)	RAM (kB)
Acinetobacter baumannii J9	3,949,341	3	yes	1:58:15	21,699,156	3,949,305	3	yes	1:39:43	16,724,392
Citrobacter koseri MINF_9D	4,832,236	3	yes	1:35:33	12,784,308	4,832,275	3	yes	2:16:14	17,732,316
Enterobacter kobei MSB1_1B	5,093,302	9	yes	1:16:42	13,362,592	5,093,446	7	yes	1:50:39	18,286,064
Haemophilus M1C132_1	2,111,116	4	yes	2:59:52	43,804,204	2,110,129	4	yes	2:35:59	18,614,324
Klebsiella oxytoca MSB1_2C	5,988,396	5	yes	1:44:27	10,231,192	5,980,979	5	yes	2:01:11	13,718,508
Klebsiella variicola INF345	5,954,241	8	yes	3:37:34	27,957,936	5,950,243	7	yes	2:20:36	16,140,824
Serratia marcescens 17-147-1671	5,816,803	5	yes	4:08:18	31,610,116	5,831,365	8	almost	2:36:58	16,137,200

Sample	Miniasm A					Miniasm B				
	Assembly size	Contigs	Complete chromosome	Time (h:m:s)	RAM (kB)	Assembly size	Contigs	Complete chromosome	Time (h:m:s)	RAM (kB)
Acinetobacter baumannii J9	4,062,256	6	yes	0:23:23	17,544,384	3,939,720	2	yes	0:14:24	13,760,300
Citrobacter koseri MINF_9D	5,500,306	10	no	0:15:25	16,014,540	8,504,708	60	no	0:32:09	19,971,672
Enterobacter kobei MSB1_1B	5,086,364	4	yes	0:08:01	8,315,212	5,164,619	8	yes	0:14:49	14,390,864
Haemophilus M1C132_1	11,432,613	52	no	0:34:10	29,268,540	12,158,170	91	yes	0:45:21	26,178,712
Klebsiella oxytoca MSB1_2C	5,984,226	4	yes	0:05:27	7,266,256	6,068,986	22	yes	0:10:39	12,093,612
Klebsiella variicola INF345	5,972,156	6	yes	0:27:25	17,238,788	5,984,371	9	yes	0:16:01	14,322,404
Serratia marcescens 17-147-1671	6,055,272	11	yes	0:21:46	21,010,204	5,922,800	5	no	0:12:12	9,911,740

Sample	Raven A					Raven B				
	Assembly size	Contigs	Complete chromosome	Time (h:m:s)	RAM (kB)	Assembly size	Contigs	Complete chromosome	Time (h:m:s)	RAM (kB)
Acinetobacter baumannii J9	3,941,649	2	yes	0:04:44	9,662,728	3,943,198	2	yes	0:03:09	7,191,816
Citrobacter koseri MINF_9D	5,375,398	13	yes	0:04:26	10,006,504	9,136,637	67	no	0:08:29	15,190,680
Enterobacter kobei MSB1_1B	5,081,245	3	yes	0:02:30	5,768,432	4,982,884	3	yes	0:04:21	9,144,468
Haemophilus M1C132_1	10,328,289	48	yes	0:08:06	16,205,452	11,595,303	68	yes	0:08:48	16,306,332
Klebsiella oxytoca MSB1_2C	5,979,704	3	yes	0:01:47	4,441,264	5,989,482	4	yes	0:02:58	7,435,080
Klebsiella variicola INF345	5,908,487	3	yes	0:06:25	13,138,252	5,968,553	6	yes	0:03:53	9,217,200
Serratia marcescens 17-147-1671	6,225,652	12	yes	0:04:43	10,020,396	5,936,778	5	no	0:03:09	6,619,112

Sample	Flye A					Flye B				
	Assembly size	Contigs	Complete chromosome	Time (h:m:s)	RAM (kB)	Assembly size	Contigs	Complete chromosome	Time (h:m:s)	RAM (kB)
Acinetobacter baumannii J9	3,943,072	2	yes	0:51:09	15,143,484	3,948,677	3	yes	0:30:09	11,995,376
Citrobacter koseri MINF_9D	4,831,828	3	yes	0:32:30	11,857,268	4,840,703	3	yes	0:50:04	16,848,976
Enterobacter kobei MSB1_1B	5,094,012	4	yes	0:23:56	10,882,488	5,096,646	5	yes	0:33:19	12,162,920
Haemophilus M1C132_1	2,132,254	4	yes	2:09:02	33,060,796	2,140,359	7	yes	2:57:43	29,133,024
Klebsiella oxytoca MSB1_2C	5,925,219	3	yes	0:20:19	10,717,304	5,993,058	5	yes	0:25:09	9,949,768
Klebsiella variicola INF345	5,978,660	6	yes	0:45:07	15,012,904	5,969,327	6	yes	0:28:25	10,974,832
Serratia marcescens 17-147-1671	5,869,366	7	yes	0:50:41	22,405,920	5,929,316	11	no	0:54:39	25,966,860

Sample	Trycycler A					Trycycler B				
	Assembly size	Contigs	Complete chromosome	Time (h:m:s)	RAM (kB)	Assembly size	Contigs	Complete chromosome	Time (h:m:s)	RAM (kB)
Acinetobacter baumannii J9	3,943,158	2	yes	1:22:21	9,293,680	3,948,814	3	yes	1:12:52	6,919,996
Citrobacter koseri MINF_9D	4,832,110	3	yes	1:20:10	8,021,512	4,831,937	3	yes	1:34:54	6,756,780
Enterobacter kobei MSB1_1B	5,081,547	3	yes	1:20:10	9,921,992	5,089,546	5	yes	1:30:33	7,316,528
Haemophilus M1C132_1	2,111,714	4	yes	1:18:17	7,354,688	2,111,568	4	yes	3:11:47	5,613,568
Klebsiella oxytoca MSB1_2C	5,925,638	3	yes	1:21:55	11,755,108	5,983,757	4	yes	1:26:13	6,906,404
Klebsiella variicola INF345	5,950,810	6	yes	1:59:29	10,954,216	5,950,424	6	yes	1:51:22	7,349,016
Serratia marcescens 17-147-1671	5,862,341	3	yes	1:53:23	11,796,584			no		

Flye circularisation

Assembly	Circ error size	Extra bases	Missing bases
Acinetobacter baumannii J9 A	-1		T
Acinetobacter baumannii J9 B	-2		AA
Citrobacter koseri MINF_9D A	0		
Citrobacter koseri MINF_9D B	-4		TAAT
Enterobacter kobei MSB1_1B A	1	T	
Enterobacter kobei MSB1_1B B	-4		AGTT
Haemophilus M1C132_1 A	0		
Haemophilus M1C132_1 B	4	TTTA	
Klebsiella oxytoca MSB1_2C A	-9		ATAGGGAGT
Klebsiella oxytoca MSB1_2C B	-1		C
Klebsiella variicola INF345 A	-4		AAAT
Klebsiella variicola INF345 B	-13		CCAGGCATCAAAT

Mean error size: 3.5833

Comparisons

Sample	Unicycler A vs B Mean identity	Unicycler A vs B Worst 100 bp identity	Miniasm A vs B Mean identity	Miniasm A vs B Worst 100 bp identity	Miniasm +Medaka A vs B Mean identity	Miniasm +Medaka A vs B Worst 100 bp identity	Miniasm +Medaka+Pilon A vs B Mean identity	Miniasm +Medaka+Pilon A vs B Worst 100 bp identity	Raven A vs B Mean identity	Raven A vs B Worst 100 bp identity	Raven +Medaka A vs B Mean identity	Raven +Medaka A vs B Worst 100 bp identity
Acinetobacter baumannii J9	99.99871%	64%	99.65311%	16%	99.73517%	33%	99.76137%	35%	99.89528%	0%	99.92994%	0%
Citrobacter koseri MINF_9D	99.99823%	28%										
Enterobacter kobei MSB1_1B	99.99731%	12%	99.93576%	49%	99.97602%	93%	99.99994%	99%	99.95272%	19%	99.97801%	19%
Haemophilus M1C132_1	99.92629%	0%							99.94055%	63%	99.99030%	78%
Klebsiella oxytoca MSB1_2C	99.85277%	11%	99.89432%	22%	99.92740%	26%	99.93321%	26%	99.96049%	19%	99.99288%	44%
Klebsiella variicola INF345	99.99500%	26%	99.93308%	0%	99.97467%	0%	99.98165%	0%	99.92892%	0%	99.96548%	0%

Sample	Raven +Medaka+Pilon A vs B Mean identity	Raven +Medaka+Pilon A vs B Worst 100 bp identity	Flye A vs B Mean identity	Flye A vs B Worst 100 bp identity	Flye +Medaka A vs B Mean identity	Flye +Medaka A vs B Worst 100 bp identity	Flye +Medaka+Pilon A vs B Mean identity	Flye +Medaka+Pilon A vs B Worst 100 bp identity	Flye (fixed circ) A vs B Mean identity	Flye (fixed circ) A vs B Worst 100 bp identity
Acinetobacter baumannii J9	99.93392%	0%	99.97512%	90%	99.99689%	98%	99.99992%	98%	99.97520%	90%
Citrobacter koseri MINF_9D			99.96541%	81%	99.99334%	96%	99.99981%	96%	99.96549%	81%
Enterobacter kobei MSB1_1B	99.99752%	19%	99.94805%	87%	99.97336%	96%	99.99971%	96%	99.94815%	87%
Haemophilus M1C132_1	99.99742%	78%	99.96929%	84%	99.99186%	96%	99.99971%	96%	99.96949%	84%
Klebsiella oxytoca MSB1_2C	99.99895%	44%	99.96640%	76%	99.99419%	91%	99.99969%	91%	99.96657%	76%
Klebsiella variicola INF345	99.97273%	0%	99.95488%	73%	99.98680%	91%	99.99978%	91%	99.95504%	73%

Sample	Flye (fixed circ) +Medaka A vs B Mean identity	Flye (fixed circ) +Medaka A vs B Worst 100 bp identity	Flye (fixed circ) +Medaka+Pilon A vs B Mean identity	Flye (fixed circ) +Medaka+Pilon A vs B Worst 100 bp identity	Trycycler A vs B Mean identity	Trycycler A vs B Worst 100 bp identity	Trycycler +Medaka A vs B Mean identity	Trycycler +Medaka A vs B Worst 100 bp identity	Trycycler +Medaka+Pilon A vs B Mean identity	Trycycler +Medaka+Pilon A vs B Worst 100 bp identity
Acinetobacter baumannii J9	99.99697%	98%	100.00000%	100%	99.98478%	97%	99.99795%	98%	100.00000%	100%
Citrobacter koseri MINF_9D	99.99342%	96%	99.99989%	99%	99.98607%	98%	99.99592%	97%	99.99998%	99%
Enterobacter kobei MSB1_1B	99.97346%	96%	99.99981%	98%	99.97128%	96%	99.98357%	97%	99.99994%	98%
Haemophilus M1C132_1	99.99206%	98%	99.99990%	99%	99.98455%	96%	99.99401%	96%	99.99976%	96%
Klebsiella oxytoca MSB1_2C	99.99437%	96%	99.99986%	96%	99.98251%	97%	99.99504%	97%	99.99998%	99%
Klebsiella variicola INF345	99.98697%	96%	99.99994%	98%	99.97923%	96%	99.99415%	97%	99.99996%	98%

ALE scores

	Unicycler	Flye	Flye +Medaka	Flye +Medaka+Pilon	Trycycler	Trycycler +Medaka	Trycycler +Medaka+Pilon
Acinetobacter baumannii J9 - read set A	-68,751,495	-69,851,511	-68,764,072	-68,670,883	-69,597,973	-68,734,531	-68,669,456
Acinetobacter baumannii J9 - read set B	-68,820,892	-70,694,877	-68,841,011	-68,673,433	-70,307,643	-68,801,834	-68,669,456
Citrobacter koseri MINF_9D - read set A	-41,431,606	-43,043,830	-41,604,961	-41,322,390	-42,440,272	-41,486,711	-41,294,467
Citrobacter koseri MINF_9D - read set B	-41,396,907	-43,306,383	-41,615,646	-41,320,183	-42,610,011	-41,508,539	-41,302,769
Enterobacter kobei MSB1_1B - read set A	-68,382,308	-70,992,926	-69,702,370	-68,368,413	-70,140,607	-69,278,458	-68,332,909
Enterobacter kobei MSB1_1B - read set B	-68,386,694	-71,141,965	-69,570,812	-68,348,288	-70,377,882	-69,242,761	-68,338,219
Haemophilus M1C132_1 - read set A	-111,530,139	-112,327,531	-111,693,172	-111,483,596	-111,951,158	-111,651,146	-111,482,933
Haemophilus M1C132_1 - read set B	-111,606,825	-112,827,118	-111,857,505	-111,492,937	-112,332,292	-111,750,901	-111,465,398
Klebsiella oxytoca MSB1_2C - read set A	-134,631,858	-137,115,849	-134,747,666	-134,454,433	-136,220,774	-134,690,431	-134,411,349
Klebsiella oxytoca MSB1_2C - read set B	-135,327,640	-137,599,430	-134,766,778	-134,420,052	-136,585,895	-134,720,644	-134,402,870
Klebsiella variicola INF345 - read set A	-82,942,439	-84,400,589	-83,230,715	-82,894,952	-83,973,858	-83,012,753	-82,892,149
Klebsiella variicola INF345 - read set B	-82,955,365	-84,664,983	-83,151,089	-82,898,292	-84,123,419	-83,038,509	-82,891,219

ALE scores (fixed circ)

	Unicycler	Flye (fixed circ)	Flye (fixed circ) +Medaka	Flye (fixed circ) +Medaka+Pilon	Trycycler	Trycycler +Medaka	Trycycler +Medaka+Pilon
Acinetobacter baumannii J9 - read set A	-68,751,495	-69,850,089	-68,762,644	-68,669,456	-69,597,973	-68,734,531	-68,669,456
Acinetobacter baumannii J9 - read set B	-68,820,892	-70,690,752	-68,837,033	-68,669,456	-70,307,643	-68,801,834	-68,669,456
Citrobacter koseri MINF_9D - read set A	-41,431,606	-43,043,830	-41,604,961	-41,322,390	-42,440,272	-41,486,711	-41,294,467
Citrobacter koseri MINF_9D - read set B	-41,396,907	-43,303,016	-41,611,806	-41,316,343	-42,610,011	-41,508,539	-41,302,769
Enterobacter kobei MSB1_1B - read set A	-68,382,308	-70,992,304	-69,700,547	-68,366,580	-70,140,607	-69,278,458	-68,332,909
Enterobacter kobei MSB1_1B - read set B	-68,386,694	-71,138,223	-69,567,063	-68,344,539	-70,377,882	-69,242,761	-68,338,219
Haemophilus M1C132_1 - read set A	-111,530,139	-112,327,531	-111,693,172	-111,483,596	-111,951,158	-111,651,146	-111,482,933
Haemophilus M1C132_1 - read set B	-111,606,825	-112,818,648	-111,848,996	-111,484,427	-112,332,292	-111,750,901	-111,465,398
Klebsiella oxytoca MSB1_2C - read set A	-134,631,858	-137,101,813	-134,733,325	-134,440,092	-136,220,774	-134,690,431	-134,411,349
Klebsiella oxytoca MSB1_2C - read set B	-135,327,640	-137,598,249	-134,765,629	-134,418,904	-136,585,895	-134,720,644	-134,402,870
Klebsiella variicola INF345 - read set A	-82,942,439	-84,398,012	-83,228,161	-82,892,397	-83,973,858	-83,012,753	-82,892,149
Klebsiella variicola INF345 - read set B	-82,955,365	-84,657,763	-83,144,016	-82,891,219	-84,123,419	-83,038,509	-82,891,219

ALE z-scores

	Unicycler	Flye	Flye +Medaka	Flye +Medaka+Pilon	Trycycler	Trycycler +Medaka	Trycycler +Medaka+Pilon
Acinetobacter baumannii J9 - read set A	0.575	-1.086	0.556	0.696	-0.704	0.600	0.699
Acinetobacter baumannii J9 - read set B	0.470	-2.360	0.440	0.693	-1.775	0.499	0.699
Citrobacter koseri MINF_9D - read set A	0.597	-1.791	0.340	0.759	-0.897	0.515	0.800
Citrobacter koseri MINF_9D - read set B	0.648	-2.180	0.324	0.762	-1.149	0.483	0.788
Enterobacter kobei MSB1_1B - read set A	0.958	-1.684	-0.378	0.972	-0.822	0.051	1.008
Enterobacter kobei MSB1_1B - read set B	0.954	-1.835	-0.245	0.993	-1.062	0.087	1.003
Haemophilus M1C132_1 - read set A	0.728	-1.288	0.316	0.845	-0.336	0.422	0.847
Haemophilus M1C132_1 - read set B	0.534	-2.551	-0.100	0.822	-1.300	0.170	0.891
Klebsiella oxytoca MSB1_2C - read set A	0.620	-1.710	0.511	0.786	-0.871	0.565	0.827
Klebsiella oxytoca MSB1_2C - read set B	-0.033	-2.164	0.493	0.818	-1.213	0.536	0.834
Klebsiella variicola INF345 - read set A	0.686	-1.698	0.215	0.764	-1.000	0.571	0.769
Klebsiella variicola INF345 - read set B	0.665	-2.130	0.345	0.759	-1.245	0.529	0.770

ALE z-scores (fixed circ)

	Unicycler	Flye (fixed circ)	Flye (fixed circ) +Medaka	Flye (fixed circ) +Medaka+Pilon	Trycycler	Trycycler +Medaka	Trycycler +Medaka+Pilon
Acinetobacter baumannii J9 - read set A	0.573	-1.087	0.556	0.697	-0.706	0.599	0.697
Acinetobacter baumannii J9 - read set B	0.468	-2.357	0.444	0.697	-1.778	0.497	0.697
Citrobacter koseri MINF_9D - read set A	0.596	-1.793	0.339	0.758	-0.899	0.514	0.799
Citrobacter koseri MINF_9D - read set B	0.647	-2.177	0.329	0.767	-1.150	0.482	0.787
Enterobacter kobei MSB1_1B - read set A	0.957	-1.685	-0.377	0.973	-0.823	0.050	1.007
Enterobacter kobei MSB1_1B - read set B	0.953	-1.833	-0.242	0.996	-1.063	0.086	1.002
Haemophilus M1C132_1 - read set A	0.725	-1.296	0.312	0.843	-0.342	0.418	0.845
Haemophilus M1C132_1 - read set B	0.531	-2.541	-0.083	0.841	-1.308	0.166	0.889
Klebsiella oxytoca MSB1_2C - read set A	0.617	-1.701	0.522	0.797	-0.874	0.562	0.824
Klebsiella oxytoca MSB1_2C - read set B	-0.036	-2.167	0.491	0.817	-1.217	0.534	0.832
Klebsiella variicola INF345 - read set A	0.684	-1.699	0.216	0.766	-1.005	0.569	0.766
Klebsiella variicola INF345 - read set B	0.663	-2.124	0.354	0.768	-1.250	0.526	0.768

IDEEL results

	Unicycler	Flye	Flye +Medaka	Flye +Medaka +Pilon	Trycycler	Trycycler +Medaka	Trycycler +Medaka +Pilon
Acinetobacter baumannii J9 - read set A	0.98074	0.83440	0.96771	0.98074	0.86345	0.97167	0.98075
Acinetobacter baumannii J9 - read set B	0.98074	0.74877	0.95873	0.98074	0.78676	0.96461	0.98075
Citrobacter koseri MINF_9D - read set A	0.97408	0.74287	0.92229	0.97363	0.79542	0.93346	0.97408
Citrobacter koseri MINF_9D - read set B	0.97385	0.70504	0.91679	0.97408	0.77871	0.93698	0.97386
Enterobacter kobei MSB1_1B - read set A	0.97649	0.71420	0.88853	0.97582	0.78683	0.93793	0.97626
Enterobacter kobei MSB1_1B - read set B	0.97649	0.67523	0.90247	0.97605	0.75682	0.93183	0.97626
Haemophilus M1C132_1 - read set A	0.95781	0.73555	0.88579	0.95829	0.83422	0.90540	0.95829
Haemophilus M1C132_1 - read set B	0.95734	0.66005	0.85060	0.95882	0.74362	0.86836	0.95833
Klebsiella oxytoca MSB1_2C - read set A	0.98601	0.74720	0.95105	0.98564	0.78714	0.95338	0.98564
Klebsiella oxytoca MSB1_2C - read set B	0.98526	0.69549	0.94823	0.98526	0.75922	0.95041	0.98583
Klebsiella variicola INF345 - read set A	0.98153	0.72098	0.90015	0.98193	0.76292	0.95594	0.98193
Klebsiella variicola INF345 - read set B	0.98193	0.69127	0.92822	0.98154	0.73622	0.94877	0.98174

IDEEL results (fixed circ)

	Unicycler	Flye (fixed circ)	Flye (fixed circ) +Medaka	Flye (fixed circ) +Medaka +Pilon	Trycycler	Trycycler +Medaka	Trycycler +Medaka +Pilon
Acinetobacter baumannii J9 - read set A	0.98074	0.83463	0.96771	0.98075	0.86345	0.97167	0.98075
Acinetobacter baumannii J9 - read set B	0.98074	0.74877	0.95873	0.98075	0.78676	0.96461	0.98075
Citrobacter koseri MINF_9D - read set A	0.97408	0.74287	0.92229	0.97363	0.79542	0.93346	0.97408
Citrobacter koseri MINF_9D - read set B	0.97385	0.70439	0.91659	0.97408	0.77871	0.93698	0.97386
Enterobacter kobei MSB1_1B - read set A	0.97649	0.71406	0.88926	0.97582	0.78683	0.93793	0.97626
Enterobacter kobei MSB1_1B - read set B	0.97649	0.67503	0.90247	0.97605	0.75682	0.93183	0.97626
Haemophilus M1C132_1 - read set A	0.95781	0.73555	0.88579	0.95829	0.83422	0.90540	0.95829
Haemophilus M1C132_1 - read set B	0.95734	0.65990	0.85060	0.95829	0.74362	0.86836	0.95833
Klebsiella oxytoca MSB1_2C - read set A	0.98601	0.74720	0.95105	0.98564	0.78714	0.95338	0.98564
Klebsiella oxytoca MSB1_2C - read set B	0.98526	0.69509	0.94840	0.98564	0.75922	0.95041	0.98583
Klebsiella variicola INF345 - read set A	0.98153	0.72086	0.89996	0.98193	0.76292	0.95594	0.98193
Klebsiella variicola INF345 - read set B	0.98193	0.69144	0.92825	0.98174	0.73622	0.94877	0.98174

Repeat identities

Sample	Unicycler A vs B				Flye A vs B				Flye +Medaka A vs B				Flye +Medaka+Pilon A vs B			
	Non-repeat identity	Non-repeat Qscore	Repeat identity	Repeat Qscore	Non-repeat identity	Non-repeat Qscore	Repeat identity	Repeat Qscore	Non-repeat identity	Non-repeat Qscore	Repeat identity	Repeat Qscore	Non-repeat identity	Non-repeat Qscore	Repeat identity	Repeat Qscore
Acinetobacter baumannii J9	99.99903%	50.1	99.98396%	37.9	99.97506%	36.0	99.97779%	36.5	99.99699%	45.2	99.99260%	41.3	100.00000%	inf	99.99630%	44.3
Citrobacter koseri MINF_9D	99.99843%	48.0	99.98941%	39.8	99.96494%	34.6	99.98652%	38.7	99.99341%	41.8	99.99037%	40.2	100.00000%	inf	99.99133%	40.6
Enterobacter kobei MSB1_1B	99.99746%	46.0	99.99088%	40.4	99.94742%	32.8	99.97537%	36.1	99.97302%	35.7	99.98814%	39.3	99.99989%	59.8	99.99179%	40.9
Haemophilus M1C132_1	99.96826%	35.0	98.02041%	17.0	99.96911%	35.1	99.97743%	36.5	99.99203%	41.0	99.98420%	38.0	100.00000%	inf	99.98646%	38.7
Klebsiella oxytoca MSB1_2C	99.85205%	28.3	99.90528%	30.2	99.96762%	34.9	99.87616%	29.1	99.99452%	42.6	99.97034%	35.3	100.00000%	inf	99.97679%	36.3
Klebsiella variicola INF345	99.99552%	43.5	99.94877%	32.9	99.95476%	33.4	99.96530%	34.6	99.98682%	38.8	99.98513%	38.3	99.99993%	61.3	99.98678%	38.8
**Average (mean)**	**99.96305%**	**34.3**	**99.57799%**	**23.7**	**99.96483%**	**34.5**	**99.95865%**	**33.8**	**99.98999%**	**40.0**	**99.98513%**	**38.3**	**99.99998%**	**66.7**	**99.98853%**	**39.4**

Sample	Flye (fixed circ) A vs B				Flye (fixed circ) +Medaka A vs B				Flye (fixed circ) +Medaka+Pilon A vs B			
	Non-repeat identity	Non-repeat Qscore	Repeat identity	Repeat Qscore	Non-repeat identity	Non-repeat Qscore	Repeat identity	Repeat Qscore	Non-repeat identity	Non-repeat Qscore	Repeat identity	Repeat Qscore
Acinetobacter baumannii J9	99.97506%	36.0	99.98149%	37.3	99.99699%	45.2	99.99630%	44.3	100.00000%	inf	100.00000%	inf
Citrobacter koseri MINF_9D	99.96494%	34.6	99.99037%	40.2	99.99341%	41.8	99.99422%	42.4	100.00000%	inf	99.99519%	43.2
Enterobacter kobei MSB1_1B	99.94750%	32.8	99.97628%	36.2	99.97310%	35.7	99.98906%	39.6	99.99998%	66.7	99.99270%	41.4
Haemophilus M1C132_1	99.96911%	35.1	99.98646%	38.7	99.99203%	41.0	99.99323%	41.7	100.00000%	inf	99.99549%	43.5
Klebsiella oxytoca MSB1_2C	99.96762%	34.9	99.88906%	29.5	99.99452%	42.6	99.98323%	37.8	100.00000%	inf	99.98968%	39.9
Klebsiella variicola INF345	99.95478%	33.4	99.97852%	36.7	99.98684%	38.8	99.99835%	47.8	99.99994%	62.5	100.00000%	inf
**Average (mean)**	**99.96485%**	**34.5**	**99.96473%**	**34.5**	**99.99001%**	**40.0**	**99.99121%**	**40.6**	**100.00000%**	**73.7**	**99.99461%**	**42.7**

Sample	Trycycler A vs B				Trycycler +Medaka A vs B				Trycycler +Medaka+Pilon A vs B			
	Non-repeat identity	Non-repeat Qscore	Repeat identity	Repeat Qscore	Non-repeat identity	Non-repeat Qscore	Repeat identity	Repeat Qscore	Non-repeat identity	Non-repeat Qscore	Repeat identity	Repeat Qscore
Acinetobacter baumannii J9	99.98491%	38.2	99.97903%	36.8	99.99793%	46.8	99.99877%	49.1	100.00000%	inf	100.00000%	inf
Citrobacter koseri MINF_9D	99.98597%	38.5	99.99037%	40.2	99.99588%	43.8	99.99807%	47.2	100.00000%	inf	99.99904%	50.2
Enterobacter kobei MSB1_1B	99.97089%	35.4	99.98814%	39.3	99.98336%	37.8	99.99270%	41.4	100.00000%	inf	99.99726%	45.6
Haemophilus M1C132_1	99.98426%	38.0	99.99774%	46.5	99.99397%	42.2	99.99549%	43.5	99.99980%	57.0	99.99774%	46.5
Klebsiella oxytoca MSB1_2C	99.98254%	37.6	99.98065%	37.1	99.99502%	43.0	99.99613%	44.1	100.00000%	inf	99.99871%	48.9
Klebsiella variicola INF345	99.97909%	36.8	99.99174%	40.8	99.99412%	42.3	99.99670%	44.8	99.99996%	64.3	100.00000%	inf
**Average (mean)**	**99.98171%**	**37.4**	**99.98718%**	**38.9**	**99.99323%**	**41.7**	**99.99623%**	**44.2**	**99.99996%**	**64.0**	**99.99855%**	**48.4**

Medaka order

Sample	Trycycler +Medaka A vs B			Medaka +Trycycler A vs B			Medaka +Trycycler +Medaka A vs B		
	Mean identity	Qscore	Worst 100 bp identity	Mean identity	Qscore	Worst 100 bp identity	Mean identity	Qscore	Worst 100 bp identity
Acinetobacter baumannii J9	99.99795%	46.88	98%	99.99710%	45.38	98%	99.99789%	46.76	98%
Citrobacter koseri MINF_9D	99.99592%	43.89	97%	99.99422%	42.38	98%	99.99523%	43.21	98%
Enterobacter kobei MSB1_1B	99.98357%	37.84	97%	99.98284%	37.65	97%	99.98334%	37.78	97%
Haemophilus M1C132_1	99.99401%	42.23	96%	99.99264%	41.33	96%	99.99381%	42.08	96%
Klebsiella oxytoca MSB1_2C	99.99504%	43.05	97%	99.99395%	42.18	98%	99.99450%	42.60	98%
Klebsiella variicola INF345	99.99415%	42.33	97%	99.99413%	42.31	97%	99.99406%	42.26	97%
