# Supplementary material for: Trycycler: consensus long-read assemblies for bacterial genomes
Source: Genome Biol. 2021 Sep 14;22:266. doi: 10.1186/s13059-021-02483-z (PMC8442456; doi:10.1186/s13059-021-02483-z)
Supplement: Supplementary file 4 — Additional file 4. Supplementary tables for the multi-user consistency tests. [file 13059_2021_2483_MOESM4_ESM.pdf]

The **Matrix** worksheet contains a presence/absence matrix for each replicon in the consistency test genomes (the same information shown in Figure 3). Cell comments are used to describe cases of misassembly and extra contigs.

The **Trycycler vs Trycycler** worksheet shows the types of differences between all pairwise combinations of Trycycler-assembled chromosomes in the consistency test genomes.

The **Trycycler vs polished** worksheet shows the types of differences between Trycycler-assembled chromosomes and the short-read-polished chromosomes in the consistency test genomes.

The **Other vs other** worksheet shows the types of differences between all pairwise combinations of single-assembler-assembled chromosomes in the consistency test genomes.

The **Tester assemblers** worksheet shows which assemblers the Trycycler testers used to generate the input assemblies for Trycycler.

Matrix

	Acinetobacter baumannii J9				*Citrobacter koseri* MINF_9D				*Enterobacter kobei* MSB1_1B						*Haemophilus* M1C132_1					*Klebsiella oxytoca* MSB1_2C					*Klebsiella variicola* INF345						
	chromosome	plasmid_1	plasmid_2	extra contigs	chromosome	plasmid_1	plasmid_2	extra contigs	chromosome	plasmid_1	plasmid_2	plasmid_3	plasmid_4	extra contigs	chromosome	plasmid_1	plasmid_2	plasmid_3	extra contigs	chromosome	plasmid_1	plasmid_2	plasmid_3	extra contigs	chromosome	plasmid_1	plasmid_2	plasmid_3	plasmid_4	plasmid_5	extra contigs
Miniasm/Minipolish	yes	yes	no	0	yes[1]	yes	yes[2]	57	yes	yes	yes	yes	yes[3]	2	yes	yes[3]	yes	yes[1]	82	yes	yes	yes	yes[3]	3	yes	yes	yes	yes	yes[3]	yes	2
Raven	yes	yes	no	0	yes[4]	yes	yes[2,3]	63	yes	yes[5]	no	no	yes[5]	0	yes	yes	yes	no	65	yes	yes	yes	yes[2]	0	yes	yes	yes	yes	yes[2]	yes[2,5]	0
Flye	yes	yes	yes	0	yes	yes	yes[2]	0	yes	yes	yes	no	yes	1	yes	yes	yes	yes	3	yes	yes	yes	yes	1	yes	yes	yes	yes[5]	yes[2]	yes	0
Trycycler - developer	yes	yes	yes	0	yes	yes	yes	0	yes	yes	yes	yes	yes	0	yes	yes	yes	yes	0	yes	yes	yes	yes	0	yes	yes	yes	yes	yes	yes	0
Trycycler - tester 1	yes	yes	yes	0	yes	yes	no	0	yes	yes	yes	yes	yes	0	yes	yes	no	yes	2	yes	yes	yes	yes	1	yes	yes	yes	no	yes	yes	0
Trycycler - tester 2	yes	yes	yes	0	yes	yes	no	0	yes	no	yes	yes	yes	0	yes	yes	yes	yes	4	yes	yes	yes	yes	1	yes	yes	yes	no	yes	yes	0
Trycycler - tester 3	yes	yes	yes	0	yes	yes	yes	0	yes	yes	yes	yes	no	0	yes	yes	no	yes	2	yes	yes	yes	yes	1	yes	yes	yes	no	yes	yes	0
Trycycler - tester 4	yes	yes	yes	0	yes	yes	yes	0	yes	yes	yes	yes	no	0	yes	yes	no	no	0	yes	yes	yes	yes	1	yes	yes	yes	yes	yes	yes	0
Trycycler - tester 5	yes	yes	yes	0	yes	yes	no	0	yes	yes	yes	yes[6]	yes[6]	0	yes	yes	no	no	3	yes	yes	yes	yes	1	yes	yes	yes	no	no	yes	0

1. Contig was too long and not circularised.
2. Multiple copies of the small plasmid sequence in one contig.
3. The assembly contains multiple redundant contigs for this plasmid, some/all of which failed to circularised well.
4. Contig was too short because some chunks of the chromosome ended up in separate contigs.
5. Contig contains misassemblies
6. The assembly contained a correct contig for this plasmid, but it also contained a misassembled contig which contains large pieces of plasmid 3 and plasmid 4.

Trycycler vs Trycycler

Genome/replicon	Replicon size	Assembly 1	Assembly 2	Substitutions	1 bp homopolymer indels	1 bp non-homopolymer indels	2 bp homopolymer indels	2 bp non-homopolymer indels	3 bp homopolymer indels	3 bp non-homopolymer indels	4 bp non-homopolymer indels
Acinetobacter_baumannii_J9_chromosome	3798645	tester_1	tester_2	4	216	8	1	0	0	0	0
Acinetobacter_baumannii_J9_chromosome	3798645	tester_1	tester_3	3	160	9	0	0	0	0	0
Acinetobacter_baumannii_J9_chromosome	3798645	tester_1	tester_4	3	212	7	0	0	0	0	0
Acinetobacter_baumannii_J9_chromosome	3798645	tester_1	tester_5	1	205	10	0	0	1	0	0
Acinetobacter_baumannii_J9_chromosome	3798645	tester_1	developer	2	271	18	0	1	0	0	0
Acinetobacter_baumannii_J9_chromosome	3798645	tester_2	tester_3	3	194	3	1	0	0	0	0
Acinetobacter_baumannii_J9_chromosome	3798645	tester_2	tester_4	3	140	3	0	0	0	0	0
Acinetobacter_baumannii_J9_chromosome	3798645	tester_2	tester_5	2	239	8	2	0	1	0	0
Acinetobacter_baumannii_J9_chromosome	3798645	tester_2	developer	4	319	12	0	1	0	0	0
Acinetobacter_baumannii_J9_chromosome	3798645	tester_3	tester_4	4	196	4	0	0	0	0	0
Acinetobacter_baumannii_J9_chromosome	3798645	tester_3	tester_5	3	210	11	1	0	0	0	0
Acinetobacter_baumannii_J9_chromosome	3798645	tester_3	developer	3	267	15	0	1	0	0	0
Acinetobacter_baumannii_J9_chromosome	3798645	tester_4	tester_5	0	258	10	1	0	1	0	0
Acinetobacter_baumannii_J9_chromosome	3798645	tester_4	developer	5	319	13	0	1	0	0	0
Acinetobacter_baumannii_J9_chromosome	3798645	tester_5	developer	4	293	12	2	1	0	0	0
Citrobacter_koseri_MINF_9D_chromosome	4758907	tester_1	tester_2	8	372	48	2	0	0	0	0
Citrobacter_koseri_MINF_9D_chromosome	4758907	tester_1	tester_3	4	211	25	1	0	0	0	0
Citrobacter_koseri_MINF_9D_chromosome	4758907	tester_1	tester_4	9	324	39	3	0	1	0	0
Citrobacter_koseri_MINF_9D_chromosome	4758907	tester_1	tester_5	4	291	46	2	0	0	0	0
Citrobacter_koseri_MINF_9D_chromosome	4758907	tester_1	developer	17	422	45	2	0	0	0	0
Citrobacter_koseri_MINF_9D_chromosome	4758907	tester_2	tester_3	8	371	47	2	0	0	0	0
Citrobacter_koseri_MINF_9D_chromosome	4758907	tester_2	tester_4	13	448	58	6	0	0	0	0
Citrobacter_koseri_MINF_9D_chromosome	4758907	tester_2	tester_5	10	398	51	1	0	0	0	0
Citrobacter_koseri_MINF_9D_chromosome	4758907	tester_2	developer	20	571	62	3	0	0	0	0
Citrobacter_koseri_MINF_9D_chromosome	4758907	tester_3	tester_4	8	312	38	2	0	0	0	0
Citrobacter_koseri_MINF_9D_chromosome	4758907	tester_3	tester_5	7	270	39	0	0	0	0	0
Citrobacter_koseri_MINF_9D_chromosome	4758907	tester_3	developer	19	375	48	0	0	0	0	0
Citrobacter_koseri_MINF_9D_chromosome	4758907	tester_4	tester_5	7	334	43	1	0	0	0	0
Citrobacter_koseri_MINF_9D_chromosome	4758907	tester_4	developer	15	445	52	1	0	0	0	0
Citrobacter_koseri_MINF_9D_chromosome	4758907	tester_5	developer	13	426	48	0	0	0	0	0
Enterobacter_kobei_MSB1_1B_chromosome	4837926	tester_1	tester_2	108	307	237	0	5	0	0	0
Enterobacter_kobei_MSB1_1B_chromosome	4837926	tester_1	tester_3	113	233	142	0	1	0	0	0
Enterobacter_kobei_MSB1_1B_chromosome	4837926	tester_1	tester_4	127	230	143	0	5	0	0	0
Enterobacter_kobei_MSB1_1B_chromosome	4837926	tester_1	tester_5	145	332	213	0	2	0	0	0
Enterobacter_kobei_MSB1_1B_chromosome	4837926	tester_1	developer	200	455	226	1	3	0	0	0
Enterobacter_kobei_MSB1_1B_chromosome	4837926	tester_2	tester_3	109	314	255	1	4	0	0	0
Enterobacter_kobei_MSB1_1B_chromosome	4837926	tester_2	tester_4	112	335	242	1	3	0	0	0
Enterobacter_kobei_MSB1_1B_chromosome	4837926	tester_2	tester_5	139	384	265	1	5	0	0	0
Enterobacter_kobei_MSB1_1B_chromosome	4837926	tester_2	developer	164	533	320	2	4	0	0	0
Enterobacter_kobei_MSB1_1B_chromosome	4837926	tester_3	tester_4	127	242	142	0	3	0	0	0
Enterobacter_kobei_MSB1_1B_chromosome	4837926	tester_3	tester_5	127	271	189	0	0	0	0	0
Enterobacter_kobei_MSB1_1B_chromosome	4837926	tester_3	developer	178	438	208	1	1	0	0	0
Enterobacter_kobei_MSB1_1B_chromosome	4837926	tester_4	tester_5	140	335	183	0	4	0	0	0
Enterobacter_kobei_MSB1_1B_chromosome	4837926	tester_4	developer	170	453	213	1	4	0	0	0
Enterobacter_kobei_MSB1_1B_chromosome	4837926	tester_5	developer	182	459	237	1	1	0	0	0
Haemophilus_M1C132_1_chromosome	2051886	tester_1	tester_2	5	172	41	0	8	0	1	0
Haemophilus_M1C132_1_chromosome	2051886	tester_1	tester_3	3	149	33	0	5	0	0	1
Haemophilus_M1C132_1_chromosome	2051886	tester_1	tester_4	9	180	44	1	11	0	0	0
Haemophilus_M1C132_1_chromosome	2051886	tester_1	tester_5	4	148	40	1	8	0	0	0
Haemophilus_M1C132_1_chromosome	2051886	tester_1	developer	4	232	53	3	10	0	0	1
Haemophilus_M1C132_1_chromosome	2051886	tester_2	tester_3	5	208	45	0	10	0	1	1
Haemophilus_M1C132_1_chromosome	2051886	tester_2	tester_4	10	218	43	0	14	0	1	0
Haemophilus_M1C132_1_chromosome	2051886	tester_2	tester_5	4	146	43	0	3	0	1	0
Haemophilus_M1C132_1_chromosome	2051886	tester_2	developer	5	238	56	1	7	0	1	1
Haemophilus_M1C132_1_chromosome	2051886	tester_3	tester_4	8	173	43	1	9	0	0	1
Haemophilus_M1C132_1_chromosome	2051886	tester_3	tester_5	1	126	26	0	10	0	0	1
Haemophilus_M1C132_1_chromosome	2051886	tester_3	developer	2	204	45	2	11	0	0	0
Haemophilus_M1C132_1_chromosome	2051886	tester_4	tester_5	6	162	34	1	11	0	0	0
Haemophilus_M1C132_1_chromosome	2051886	tester_4	developer	8	212	47	6	14	0	0	1
Haemophilus_M1C132_1_chromosome	2051886	tester_5	developer	1	186	39	2	7	0	0	1
Klebsiella_oxytoca_MSB1_2C_chromosome	5804453	tester_1	tester_2	3	289	8	1	1	0	0	0
Klebsiella_oxytoca_MSB1_2C_chromosome	5804453	tester_1	tester_3	7	478	9	3	2	0	0	0
Klebsiella_oxytoca_MSB1_2C_chromosome	5804453	tester_1	tester_4	5	325	5	0	2	0	0	0
Klebsiella_oxytoca_MSB1_2C_chromosome	5804453	tester_1	tester_5	7	367	12	1	1	0	0	0
Klebsiella_oxytoca_MSB1_2C_chromosome	5804453	tester_1	developer	5	490	9	1	4	0	0	0
Klebsiella_oxytoca_MSB1_2C_chromosome	5804453	tester_2	tester_3	6	403	9	0	1	0	0	0
Klebsiella_oxytoca_MSB1_2C_chromosome	5804453	tester_2	tester_4	1	245	8	1	1	0	0	0
Klebsiella_oxytoca_MSB1_2C_chromosome	5804453	tester_2	tester_5	6	302	8	1	0	0	0	0
Klebsiella_oxytoca_MSB1_2C_chromosome	5804453	tester_2	developer	4	437	11	3	3	0	0	0
Klebsiella_oxytoca_MSB1_2C_chromosome	5804453	tester_3	tester_4	6	369	4	1	2	0	0	0
Klebsiella_oxytoca_MSB1_2C_chromosome	5804453	tester_3	tester_5	3	389	9	1	1	0	0	0
Klebsiella_oxytoca_MSB1_2C_chromosome	5804453	tester_3	developer	6	518	8	2	4	0	0	0
Klebsiella_oxytoca_MSB1_2C_chromosome	5804453	tester_4	tester_5	8	322	9	1	1	0	0	0
Klebsiella_oxytoca_MSB1_2C_chromosome	5804453	tester_4	developer	6	433	6	3	4	0	0	0
Klebsiella_oxytoca_MSB1_2C_chromosome	5804453	tester_5	developer	3	429	9	3	3	0	0	0
Klebsiella_variicola_INF345_chromosome	5417034	tester_1	tester_2	25	260	17	0	0	0	0	0
Klebsiella_variicola_INF345_chromosome	5417034	tester_1	tester_3	22	537	22	1	4	0	0	0
Klebsiella_variicola_INF345_chromosome	5417034	tester_1	tester_4	20	505	33	0	2	0	0	0
Klebsiella_variicola_INF345_chromosome	5417034	tester_1	tester_5	21	563	33	2	6	0	0	0
Klebsiella_variicola_INF345_chromosome	5417034	tester_1	developer	24	532	37	0	7	0	0	0
Klebsiella_variicola_INF345_chromosome	5417034	tester_2	tester_3	20	549	27	1	4	0	0	0
Klebsiella_variicola_INF345_chromosome	5417034	tester_2	tester_4	28	550	31	0	3	0	0	0
Klebsiella_variicola_INF345_chromosome	5417034	tester_2	tester_5	20	610	29	2	7	0	0	0
Klebsiella_variicola_INF345_chromosome	5417034	tester_2	developer	31	556	36	0	6	0	0	0
Klebsiella_variicola_INF345_chromosome	5417034	tester_3	tester_4	15	420	19	1	3	0	0	0
Klebsiella_variicola_INF345_chromosome	5417034	tester_3	tester_5	6	389	16	2	4	0	0	0
Klebsiella_variicola_INF345_chromosome	5417034	tester_3	developer	20	620	20	0	5	0	0	0
Klebsiella_variicola_INF345_chromosome	5417034	tester_4	tester_5	16	479	29	3	4	0	0	0
Klebsiella_variicola_INF345_chromosome	5417034	tester_4	developer	29	645	30	0	5	0	0	0
Klebsiella_variicola_INF345_chromosome	5417034	tester_5	developer	23	626	25	1	4	0	0	0

**Totals:**	**2818**	**30781**	**5207**	**96**	**282**	**4**	**5**	**8**
**Proportions:**	**7.19%**	**78.52%**	**13.28%**	**0.24%**	**0.72%**	**0.01%**	**0.01%**	**0.02%**
**Rate per Mbp:**	**6.63**	**77.55**	**13.90**	**0.27**	**1.05**	**0.01**	**0.03**	**0.04**

Trycycler vs polished

Genome/replicon	Replicon size	Assembly 1	Assembly 2	Substitutions	1 bp homopolymer indels	1 bp non-homopolymer indels	2 bp homopolymer indels	2 bp non-homopolymer indels	3 bp homopolymer indels	3 bp non-homopolymer indels	4 bp homopolymer indels	4 bp non-homopolymer indels	5 bp homopolymer indels	5 bp non-homopolymer indels	6 bp non-homopolymer indels	7 bp non-homopolymer indels	8 bp non-homopolymer indels	9 bp non-homopolymer indels	12 bp non-homopolymer indels	13 bp non-homopolymer indels	16 bp non-homopolymer indels	methylation motif errors	non-methylation motif errors
Acinetobacter_baumannii_J9_chromosome	3798645	tester_1	polished	2	764	6	27	0	2	0	0	0	0	0	0	0	0	0	0	0	0	1	7
Acinetobacter_baumannii_J9_chromosome	3798645	tester_2	polished	3	734	4	23	0	2	0	0	0	0	0	0	0	0	0	0	0	0	0	7
Acinetobacter_baumannii_J9_chromosome	3798645	tester_3	polished	5	728	6	26	0	1	0	0	0	0	0	0	0	0	0	0	0	0	1	10
Acinetobacter_baumannii_J9_chromosome	3798645	tester_4	polished	1	713	4	22	0	2	0	0	0	0	0	0	0	0	0	0	0	0	0	5
Acinetobacter_baumannii_J9_chromosome	3798645	tester_5	polished	0	727	8	23	0	1	0	0	0	0	0	0	0	0	0	0	0	0	1	7
Acinetobacter_baumannii_J9_chromosome	3798645	developer	polished	3	845	14	33	1	0	0	0	0	0	0	0	0	0	0	0	0	0	1	17
Citrobacter_koseri_MINF_9D_chromosome	4758907	tester_1	polished	10	1201	81	68	1	11	0	0	0	0	0	0	0	0	0	0	0	0	1	91
Citrobacter_koseri_MINF_9D_chromosome	4758907	tester_2	polished	12	1206	99	61	1	8	0	0	0	0	0	0	0	0	0	0	0	0	1	111
Citrobacter_koseri_MINF_9D_chromosome	4758907	tester_3	polished	9	1094	86	59	1	9	0	0	0	0	0	0	0	0	0	0	0	0	1	95
Citrobacter_koseri_MINF_9D_chromosome	4758907	tester_4	polished	14	1096	80	63	1	7	0	0	0	0	0	0	0	0	0	0	0	0	1	94
Citrobacter_koseri_MINF_9D_chromosome	4758907	tester_5	polished	13	1170	73	62	1	7	0	0	0	0	0	0	0	0	0	0	0	0	1	86
Citrobacter_koseri_MINF_9D_chromosome	4758907	developer	polished	15	1068	76	61	1	4	0	0	0	0	0	0	0	0	0	0	0	0	1	91
Enterobacter_kobei_MSB1_1B_chromosome	4837926	tester_1	polished	299	1284	540	83	2	4	0	1	0	0	0	0	0	0	0	0	0	0	0	841
Enterobacter_kobei_MSB1_1B_chromosome	4837926	tester_2	polished	223	1326	582	88	4	5	0	0	0	1	0	0	0	0	0	0	0	0	0	809
Enterobacter_kobei_MSB1_1B_chromosome	4837926	tester_3	polished	318	1303	537	82	0	3	0	1	0	0	0	0	0	0	0	0	0	0	0	855
Enterobacter_kobei_MSB1_1B_chromosome	4837926	tester_4	polished	293	1251	543	91	5	5	0	1	0	0	0	0	0	0	0	0	0	0	0	841
Enterobacter_kobei_MSB1_1B_chromosome	4837926	tester_5	polished	283	1368	573	90	0	4	0	1	0	0	0	0	0	0	0	0	0	0	0	856
Enterobacter_kobei_MSB1_1B_chromosome	4837926	developer	polished	284	1352	599	78	1	7	0	0	0	1	0	0	0	0	0	0	0	0	1	883
Haemophilus_M1C132_1_chromosome	2051886	tester_1	polished	3	432	43	15	15	2	0	0	1	0	0	0	0	0	0	0	0	0	0	62
Haemophilus_M1C132_1_chromosome	2051886	tester_2	polished	4	425	54	17	6	3	1	0	1	0	0	0	0	0	0	0	0	0	0	66
Haemophilus_M1C132_1_chromosome	2051886	tester_3	polished	1	372	41	16	21	2	0	0	0	0	0	0	0	0	0	0	0	0	0	63
Haemophilus_M1C132_1_chromosome	2051886	tester_4	polished	6	388	53	14	23	2	0	0	1	0	0	0	0	0	0	0	0	0	0	83
Haemophilus_M1C132_1_chromosome	2051886	tester_5	polished	0	381	47	15	9	1	0	0	1	0	0	0	0	0	0	0	0	0	0	57
Haemophilus_M1C132_1_chromosome	2051886	developer	polished	1	407	40	17	10	3	0	0	0	0	0	0	0	0	0	0	0	0	0	51
Klebsiella_oxytoca_MSB1_2C_chromosome	5804453	tester_1	polished	4	1738	18	118	5	7	0	1	0	0	0	0	0	0	0	0	0	0	3	24
Klebsiella_oxytoca_MSB1_2C_chromosome	5804453	tester_2	polished	2	1611	14	104	4	7	0	0	0	0	0	0	0	0	0	0	0	0	3	17
Klebsiella_oxytoca_MSB1_2C_chromosome	5804453	tester_3	polished	2	1612	16	95	3	8	0	0	0	0	0	0	0	0	0	0	0	0	3	18
Klebsiella_oxytoca_MSB1_2C_chromosome	5804453	tester_4	polished	2	1562	15	99	3	6	0	0	0	0	0	0	0	0	0	0	0	0	3	17
Klebsiella_oxytoca_MSB1_2C_chromosome	5804453	tester_5	polished	4	1554	9	102	4	7	0	0	0	0	0	0	0	0	0	0	0	0	2	15
Klebsiella_oxytoca_MSB1_2C_chromosome	5804453	developer	polished	4	1568	13	98	7	7	0	1	0	0	0	0	0	0	0	0	0	0	3	21
Klebsiella_variicola_INF345_chromosome	5417034	tester_1	polished	22	1967	44	110	13	9	5	1	8	1	2	4	2	3	3	1	1	1	1	108
Klebsiella_variicola_INF345_chromosome	5417034	tester_2	polished	22	2005	47	124	14	7	5	1	8	1	2	4	2	3	3	1	1	1	2	111
Klebsiella_variicola_INF345_chromosome	5417034	tester_3	polished	5	1632	26	89	10	7	5	0	8	1	2	4	2	3	3	1	1	1	0	71
Klebsiella_variicola_INF345_chromosome	5417034	tester_4	polished	13	1720	42	91	11	4	5	0	8	1	2	4	2	3	3	1	1	1	0	96
Klebsiella_variicola_INF345_chromosome	5417034	tester_5	polished	5	1604	38	88	12	7	5	1	8	0	2	4	2	3	3	1	1	1	2	83
Klebsiella_variicola_INF345_chromosome	5417034	developer	polished	21	1945	38	118	12	8	5	0	8	1	2	4	2	3	3	1	1	1	0	101
			**Totals:**	**1908**	**42153**	**4509**	**2370**	**201**	**179**	**31**	**9**	**52**	**7**	**12**	**24**	**12**	**18**	**18**	**6**	**6**	**6**	**33**	**6770**
			**Proportions:**	**3.70%**	**81.82%**	**8.75%**	**4.60%**	**0.39%**	**0.35%**	**0.06%**	**0.02%**	**0.10%**	**0.01%**	**0.02%**	**0.05%**	**0.02%**	**0.03%**	**0.03%**	**0.01%**	**0.01%**	**0.01%**	**0.49%**	**99.51%**
			**Rate per Mbp:**	**11.03**	**252.62**	**27.94**	**13.65**	**1.74**	**1.08**	**0.17**	**0.05**	**0.30**	**0.04**	**0.06**	**0.12**	**0.06**	**0.09**	**0.09**	**0.03**	**0.03**	**0.03**	**0.18**	**41.53**

Other vs other

Genome/replicon	Replicon size	Assembly 1	Assembly 2	Substitutions	1 bp homopolymer indels	1 bp non-homopolymer indels	2 bp homopolymer indels	2 bp non-homopolymer indels	3 bp homopolymer indels	3 bp non-homopolymer indels	4 bp homopolymer indels	4 bp non-homopolymer indels	5 bp non-homopolymer indels	6 bp non-homopolymer indels	7 bp non-homopolymer indels	8 bp non-homopolymer indels	9 bp non-homopolymer indels	10 bp non-homopolymer indels	11 bp non-homopolymer indels	12 bp non-homopolymer indels	13 bp non-homopolymer indels	14 bp non-homopolymer indels	15 bp non-homopolymer indels	16 bp non-homopolymer indels	17 bp non-homopolymer indels	18 bp non-homopolymer indels	19 bp non-homopolymer indels	20 bp non-homopolymer indels	21 bp non-homopolymer indels	22 bp non-homopolymer indels	25 bp non-homopolymer indels	26 bp non-homopolymer indels	32 bp non-homopolymer indels	91 bp non-homopolymer indels	106 bp non-homopolymer indels	458 bp non-homopolymer indels
Acinetobacter_baumannii_J9_chromosome	3798645	miniasm	raven	2975	670	2507	67	769	8	283	3	148	82	41	22	20	14	10	8	6	7	4	1	1	0	1	3	2	0	1	0	0	0	1	0	1
Acinetobacter_baumannii_J9_chromosome	3798645	miniasm	flye	2950	968	2335	64	724	8	247	2	133	60	34	16	14	9	4	4	2	3	4	1	1	0	1	1	0	0	0	0	0	0	0	1	0
Acinetobacter_baumannii_J9_chromosome	3798645	raven	flye	48	804	243	19	88	1	51	1	41	22	16	13	12	10	4	5	1	2	2	2	1	0	0	1	0	3	0	1	0	0	0	0	0
Enterobacter_kobei_MSB1_1B_chromosome	4837926	miniasm	raven	215	692	1074	36	227	2	56	2	17	5	0	0	1	1	0	0	0	0	1	0	0	0	0	0	0	0	0	0	0	0	0	0	0
Enterobacter_kobei_MSB1_1B_chromosome	4837926	miniasm	flye	414	1159	1037	37	155	2	41	2	12	5	0	0	0	1	0	0	0	0	1	0	0	0	0	0	0	0	0	0	0	0	0	0	0
Enterobacter_kobei_MSB1_1B_chromosome	4837926	raven	flye	307	1101	1008	19	99	2	10	0	7	0	0	0	1	0	0	0	0	0	0	0	0	0	0	0	0	0	0	0	0	0	0	0	0
Haemophilus_M1C132_1_chromosome	2051886	miniasm	raven	120	322	431	66	166	9	48	0	7	3	2	2	2	0	0	0	0	1	0	0	0	0	0	0	0	0	0	0	0	0	0	0	0
Haemophilus_M1C132_1_chromosome	2051886	miniasm	flye	107	514	326	43	106	10	28	1	3	1	0	0	0	0	0	0	0	0	0	0	0	0	0	0	0	0	0	0	0	0	0	0	0
Haemophilus_M1C132_1_chromosome	2051886	raven	flye	77	480	269	23	99	2	25	0	6	4	2	2	2	0	0	0	0	1	0	0	0	0	0	0	0	0	0	0	0	0	0	0	0
Klebsiella_oxytoca_MSB1_2C_chromosome	5804453	miniasm	raven	796	713	1208	37	493	4	219	1	102	61	33	22	12	15	9	0	3	2	2	1	0	1	0	0	0	0	0	0	1	0	0	0	0
Klebsiella_oxytoca_MSB1_2C_chromosome	5804453	miniasm	flye	842	1400	1108	47	450	4	207	1	100	56	31	19	9	15	8	0	3	2	1	1	0	1	0	0	0	0	0	0	0	0	0	0	0
Klebsiella_oxytoca_MSB1_2C_chromosome	5804453	raven	flye	71	1348	243	31	92	0	17	0	1	8	0	2	2	1	0	0	0	0	1	0	0	0	0	0	0	0	0	0	1	1	0	0	0
Klebsiella_variicola_INF345_chromosome	5417034	miniasm	raven	131	805	594	40	179	2	40	0	11	4	0	3	0	0	0	0	0	0	0	0	0	0	0	0	0	0	0	1	0	0	0	0	0
Klebsiella_variicola_INF345_chromosome	5417034	miniasm	flye	285	1451	622	35	156	1	29	0	6	2	0	1	0	0	0	0	0	0	0	0	0	0	0	0	0	0	0	0	0	0	0	0	0
Klebsiella_variicola_INF345_chromosome	5417034	raven	flye	244	1381	454	32	107	0	21	0	6	4	0	4	0	0	0	0	0	0	0	0	0	0	0	0	0	0	0	1	0	0	0	0	0
			**Totals:**	**9582**	**13808**	**13459**	**596**	**3910**	**55**	**1322**	**13**	**600**	**317**	**159**	**106**	**75**	**66**	**35**	**17**	**15**	**18**	**16**	**6**	**3**	**2**	**2**	**5**	**2**	**3**	**1**	**3**	**2**	**1**	**1**	**1**	**1**
			**Proportions:**	**21.68%**	**31.24%**	**30.45%**	**1.35%**	**8.85%**	**0.12%**	**2.99%**	**0.03%**	**1.36%**	**0.72%**	**0.36%**	**0.24%**	**0.17%**	**0.15%**	**0.08%**	**0.04%**	**0.03%**	**0.04%**	**0.04%**	**0.01%**	**0.01%**	**0.00%**	**0.00%**	**0.01%**	**0.00%**	**0.01%**	**0.00%**	**0.01%**	**0.00%**	**0.00%**	**0.00%**	**0.00%**	**0.00%**
			**Rate per Mbp:**	**155.35**	**210.80**	**215.50**	**10.83**	**63.76**	**1.19**	**21.15**	**0.22**	**9.28**	**4.83**	**2.46**	**1.62**	**1.23**	**0.96**	**0.51**	**0.30**	**0.23**	**0.32**	**0.25**	**0.09**	**0.05**	**0.02**	**0.04**	**0.09**	**0.04**	**0.05**	**0.02**	**0.04**	**0.02**	**0.01**	**0.02**	**0.02**	**0.02**

Tester assemblers

	Assemblers used
Trycycler - developer	3× Flye, 3× Miniasm/Minipolish, 3× Raven, 3× Redbean
Trycycler - tester 1	3× Flye, 3× Miniasm/Minipolish, 3× Raven, 3× Redbean
Trycycler - tester 2	3× Flye, 3× Miniasm/Minipolish, 3× Raven, 3× Redbean
Trycycler - tester 3	4× Flye, 4× Miniasm/Minipolish, 4× Raven
Trycycler - tester 4	3× Flye, 3× Miniasm/Minipolish, 3× Raven
Trycycler - tester 5	4× Flye, 4× Miniasm/Minipolish, 4× Raven
