# Supplementary material for: Trycycler: consensus long-read assemblies for bacterial genomes
Source: Genome Biol. 2021 Sep 14;22:266. doi: 10.1186/s13059-021-02483-z (PMC8442456; doi:10.1186/s13059-021-02483-z)
Supplement: Supplementary file 5 — Additional file 5. Review history. [file 13059_2021_2483_MOESM5_ESM.docx]

Review History

**First round of review**

**Reviewer 1**

**Were you able to assess all statistics in the manuscript, including the appropriateness of statistical tests used?**

There are no statistics in the manuscript.

**Were you able to directly test the methods?**

Yes

**Comments to author:**

The manuscript describes an approach to generate reference-quality bacterial genomes called Trycycler. Given a set of (imperfect) long-read assemblies as input, Trycycler produces a consensus assembly of a higher quality, leveraging the fact that the long-read assembly algorithms often make different errors in different genome locations. The authors evaluate Trycycler using several bacterial genomes using both simulated and real sequencing data and conclude that it fixes the most artifacts of the existing long-read assemblers and produces high-quality bacterial genomes.

The paper is clearly written, very detailed and interesting to read. The authors correctly note that even though the current state-of-the-art assemblers recover complete bacterial chromosomes, the assemblies often contain artifacts that need to be corrected. Trycycler helps to solve this issue and will be a valuable tool for many bacterial genome sequencing projects.

The authors are making an interesting choice of making the pipeline semi-automated, as Trycycler requires judgement of a human expert at certain stages. With respect to that, I only have a minor suggestion of expanding the discussion on that topic. How difficult (if at all possible) would it be to make the pipeline fully automatic? Or alternatively, can the pipeline make "suggestions" for a human expert to review and either accept or intervene? What does a typical human interaction look like? I found some illustrative examples of that in the Trycycler documentation, but I think the manuscript could benefit from including some detail as well.

**Reviewer 2**

**Were you able to assess all statistics in the manuscript, including the appropriateness of statistical tests used?**

Yes, and I have assessed the statistics in my report.

**Were you able to directly test the methods?**

Yes

**Comments to author:**

Trycycler is a consensus assembly tool coupled with human skill and interaction. (though arguably this is always the case, though often overlooked). It requires a deep dataset, which it subsamples, assembles with multiple assemblers (ideal), and then creates a consensus assembly through means of clustering, re-organisation, multiple sequence alignment, human QC, and error correction. Finally these consensus assemblies are polished with medaka (for ONT) and pilon (if short read data available). The results show Trycycler outperforms regular assemblers both with simulated and real reads.

Overall Comments:

This has honestly been the best written paper I have reviewed to date, and an enjoyable paper to read. I found myself formulating questions, only to have them thoroughly answered in the next paragraph (such as Flye almost always having the start/end site occur in a repetitive element, which also ended up having errors). The documentation alongside the code on github is excellent, and a demonstration of "good" documentation that all bioinformatic developers should aspire to. I ran trycycler myself, and found it a little intimidating at first, but by following the documentation and using the example data, I successfully created some very nice assemblies. It was a little tricky in the QC step, and removing directories was a little daunting, and a little irregular, but as a dev myself, I can't think of a better way to approach that which doesn't overly complicate the workflow (and code).

Major:

I have no major edits or concerns about the manuscript. This work would be a valuable addition to the literature in the field of bacterial isolate genome assembly.

Minor:

The authors show a workflow of:

assemblies > trycycler > medaka, pilon

Could a user do:

assemblies > medaka on each > trycycler > medaka (optional) > pilon

or even:

assemblies > medaka on each > pilon on each > trycycler > medaka (optional) > pilon (optional)

for even better results? Or are there considerations other than time and repetition to not do this? It would be useful to know, using the samples used in this manuscript, what impact, if any, these methods would yield. (I know this is time consuming, sorry).

Though trycycler will tend towards the true sequence of assembly, it will still tend towards systematic errors in the sequencing platform output. These could be homopolymer regions, or difficult motifs to sequence for ONT or Pac Bio. Was there any indication in the final assemblies of these systematic errors "bleeding through", and were there any common motifs which tended to give more trouble than others in resolving clean sequences? Further to that, if a sample contains native DNA, were errors around base modifications increased, or not a factor? A comment on systematic error and tending towards it with trycycler would be useful to have in the discussion of the manuscript.

**Authors Response**

**Point-by-point responses to the reviewers’ comments:**

Reviewer 1

1. The authors are making an interesting choice of making the pipeline semi-automated, as Trycycler requires judgement of a human expert at certain stages. With respect to that, I only have a minor suggestion of expanding the discussion on that topic. How difficult (if at all possible) would it be to make the pipeline fully automatic? Or alternatively, can the pipeline make "suggestions" for a human expert to review and either accept or intervene? What does a typical human interaction look like? I found some illustrative examples of that in the Trycycler documentation, but I think the manuscript could benefit from including some detail as well.

*We have added the following text in the ‘Approach and implementation’ section describing the Trycycler reconciliation step:*

*"Cluster reconciliation will fail if a contig cannot be circularised or if any of the pairwise alignments within the cluster have low identity. In such cases, Trycycler will suggest interventions to resolve the issue, but it is up to the user to manually exclude or modify the contig sequences as necessary."*

*We have also expanded on the topics of automation and manual intervention in the Discussion, including the addition of a new supplementary figure which gives examples of the process:*

*"Creating a Trycycler assembly often requires judgement calls and manual intervention, particularly after Trycycler’s clustering step where users must decide which contig clusters are valid (represent true replicons in the genome), which clusters are invalid (spurious, misassembled or contaminant sequences) and whether any contig sequences need to be trimmed or excluded to allow for cluster reconciliation. While fully automated pipelines are useful for high-throughput analyses, this would be difficult and undesirable for Trycycler. There are many ways that long-read assembly can fail, and Trycycler does not restrict which input assemblers can be used, so planning for all failure modes is not possible. Trycycler also allows users to subjectively judge the ‘assemblability’ of their read set based on the coherence of the contig clustering. If input contigs cluster well, users can proceed with confidence. If not, they should investigate why the reads failed to produce consistent assemblies, e.g. insufficient read length or genome heterogeneity. See Additional file 1: Fig. S7 for examples of this process in the real read tests."*

Reviewer 2

1. The authors show a workflow of: assemblies > trycycler > medaka, pilon. Could a user do: assemblies > medaka on each > trycycler > medaka (optional) > pilon, or even: assemblies > medaka on each > pilon on each > trycycler > medaka (optional) > pilon (optional) for even better results? Or are there considerations other than time and repetition to not do this? It would be useful to know, using the samples used in this manuscript, what impact, if any, these methods would yield. (I know this is time consuming, sorry).

*We have tried running different combinations of running Medaka and Trycycler, and have added the following text to the Results section:*

*"While the above results used Medaka polishing after Trycycler (i.e. Trycycler+Medaka), it is also possible to run Medaka polishing on Trycycler’s input assemblies (i.e. Medaka+Trycycler) or on both Trycycler’s input assemblies and its final assembly (i.e. Medaka+Trycycler+Medaka). We tried these alternative approaches using the real read data and found that while all performed similarly (mean identity Q41–Q42), the best results were achieved when Medaka was the final step in the process (Additional file 3: Medaka order). We therefore recommend the Trycycler+Medaka approach both for its simplicity and accuracy."*

*More combinations are possible (by integrating Pilon into the process), however a deep exploration of short-read polishing techniques falls outside the scope of this paper.*

2. Though Trycycler will tend towards the true sequence of assembly, it will still tend towards systematic errors in the sequencing platform output. These could be homopolymer regions, or difficult motifs to sequence for ONT or Pac Bio. Was there any indication in the final assemblies of these systematic errors "bleeding through", and were there any common motifs which tended to give more trouble than others in resolving clean sequences? Further to that, if a sample contains native DNA, were errors around base modifications increased, or not a factor? A comment on systematic error and tending towards it with Trycycler would be useful to have in the discussion of the manuscript.

*We have added a new paragraph in the Discussion which more thoroughly addresses the concept of systematic basecalling errors and their relation to Trycycler assembly accuracy:*

*"Even ideal Trycycler assemblies still contain small-scale errors. Our real read tests achieved an accuracy of Q37, equivalent to about one error per 5 kbp of sequence, most of which (>80%, see Additional file 4: Trycycler vs polished) were in homopolymer sequences. While we have previously shown that many errors in a long-read Klebsiella pneumoniae assembly occurred in Dcm methylation motifs, this was not the case for genomes in this study (Additional file 4: Trycycler vs polished). Residual small-scale errors in assemblies result from systematic basecalling errors (i.e. when many of the reads covering a genomic position contain the same error) and therefore cannot be entirely avoided in the assembly process. Instead, these errors can be addressed before assembly (during basecalling) or after assembly (with polishing). Superior ONT basecalling can be achieved with improvements in sequencing chemistry, neural networks and training sets, so future developments in these areas will result in better Trycycler assembly accuracy."*
